# Control of Scar Tissue Formation in the Cornea: Strategies in Clinical and Corneal Tissue Engineering

**DOI:** 10.3390/jfb3030642

**Published:** 2012-09-18

**Authors:** Samantha L. Wilson, Alicia J. El Haj, Ying Yang

**Affiliations:** Institute for Science and Technology in Medicine, School of Medicine, Keele University, Staffordshire, ST4 7QB, UK; Email: s.l.wilson@ keele.ac.uk (S.L.W.); a.j.el.haj@ keele.ac.uk (A.J.E.H.)

**Keywords:** cornea, keratocyte, fibroblast, differentiation, scarring, disease

## Abstract

Corneal structure is highly organized and unified in architecture with structural and functional integration which mediates transparency and vision. Disease and injury are the second most common cause of blindness affecting over 10 million people worldwide. Ninety percent of blindness is permanent due to scarring and vascularization. Scarring caused via fibrotic cellular responses, heals the tissue, but fails to restore transparency. Controlling keratocyte activation and differentiation are key for the inhibition and prevention of fibrosis. Ophthalmic surgery techniques are continually developing to preserve and restore vision but corneal regression and scarring are often detrimental side effects and long term continuous follow up studies are lacking or discouraging. Appropriate corneal models may lead to a reduced need for corneal transplantation as presently there are insufficient numbers or suitable tissue to meet demand. Synthetic optical materials are under development for keratoprothesis although clinical use is limited due to implantation complications and high rejection rates. Tissue engineered corneas offer an alternative which more closely mimic the morphological, physiological and biomechanical properties of native corneas. However, replication of the native collagen fiber organization and retaining the phenotype of stromal cells which prevent scar-like tissue formation remains a challenge. Careful manipulation of culture environments are under investigation to determine a suitable environment that simulates native ECM organization and stimulates keratocyte migration and generation.

## 1. The Importance and Correlation of Transparency and Scar Tissue

Vision is reliant upon the discrete organization, structure and functional integration [1] of the corneal stroma and its components. Changes in corneal shape and structure that disrupt the complex organization cause scarring and opacities and are often the result of the majority of corneal diseases and injury [2,3]. Often corneal blindness is the result of corneal scarring and vascularization which if severe is permanent. The characteristics of scar tissue is an abnormal alignment of the collagen fibrils, which is directly associated with transparency of the tissue. The impact of scar tissue formation in corneal tissue is significantly greater than in other tissue types as it has direct impacts on vision. Understanding the *in vivo* mechanisms whereby the cornea is able to regenerate rather than repair is fundamental to clinical approaches as it often determines the difference between a functioning transparent cornea and an opaque scarred tissue. 

**Figure 1:**
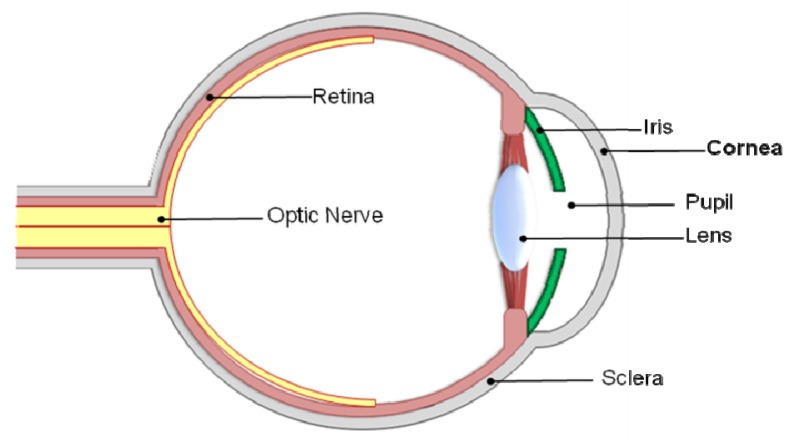
Basic anatomy of the human eye and location of the cornea.

The cornea comprises one-sixth of the total ocular globe [1] (Figure 1) with a structure described as “*the epitome of efficiently unified structure and function*” [4] due to its complexity and extracellular organization. The avascular [5,6], sparsely populated, viscoeleastic [7,8], multilaminar structure [9] forms a barrier to protect the intraocular contents, is transparent to visible light and forms an almost perfect optical boundary to refract light onto the retina [9], providing two-thirds of the optical power of the eye [10]. 

**Figure 2:**
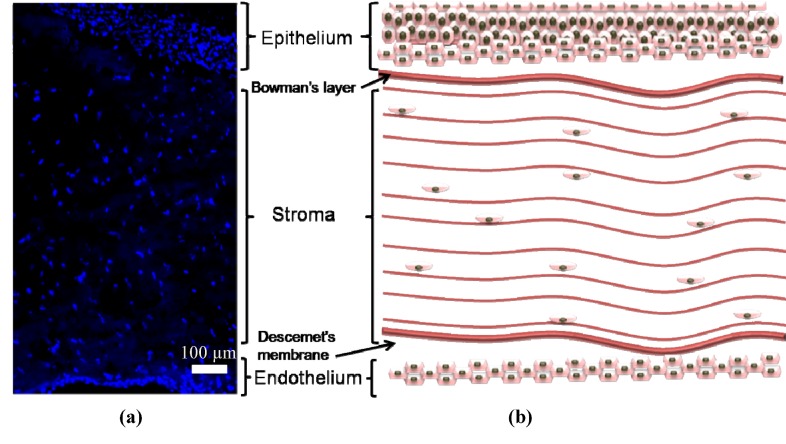
(**a**) DAPI stained cross section of the cornea detailing the 3 distinct cellular layers of the cornea, E, Alicia J. El Haj pithelium, Stroma and Endothelium; (**b**) schematic representation of the corneal layers with additional Bowman’s layer and Descemet’s membrane detailed.

The corneal tissue consists of five distinct layers: Epithelial; Bowman’s; Stromal; Descemets; and Endothelial layers with the three cellular functional layers being the epithelium, stroma and the endothelium (Figure 2). A healthy epithelium has 5–7 cellular layers with 3 distinct highly differentiated and self-renewing cell types; the strata germinatum; daughter/wing cells; and squamous cells [11]. The epithelium’s main role is to protect the stroma from invasion by restricting foreign material entering the eye. The bowman’s layer is a transparent sheet of tissue residing just below the basement membrane of the epithelium. It is less complex in comparison to the other layers with randomly arranged collagen fibers [12]. It scars when damaged, resulting in vision loss if the scars are large and centrally located [13]. The stromal layer occupies approximately 90% of the total thickness of the cornea and has the most organized extracellular (ECM) in the body [13]. The stroma is responsible for the overall strength and shape of the cornea [3]. Disruption to the stromal layer by disease or injury is often the cause of permanent blindness. The descemets membrane is a thin but strong barrier against infection and injury that aids in maintaining corneal curvature [8,14]. It is derived from secretions from endothelial cells [8,15] and is able to regenerate after infection. The endothelium is the very thin, innermost layer of the cornea. It is made up of closely interdigitated cells arranged in a mosaic pattern of mostly hexagonal shapes [11]. It is essential for keeping the cornea clear as it pumps excess fluid out of the stroma. This prevents stromal swelling, hazing and ultimately the occurrence of an opaque stromal layer. Endothelial cell loss results in enlargement and spread of neighbouring cells to cover the defective area [11]. Therefore, damage to the endothelial cells results in permanent destruction which leads to corneal edema (swelling) and blindness. 

**Figure 3:**
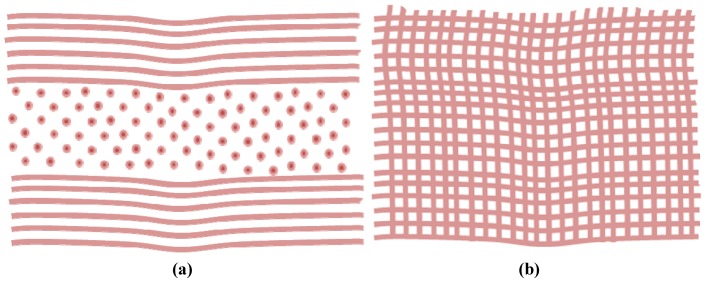
A schematic representation of (**a**) a cross sectional view; (**b**) a top view of the microstructural arrangement of the collagen fibrils: the collagen fibrils within the stromal layer are arranged parallel to each other in regularly spaced lamellae sheets.

Corneal transparency is heavily dependent upon the highly complex levels of organization and regular spatial order of the thin collagen fibrils within the stromal layer [3,16,17]. The collagen fibrils are approximately 25–35 nm [16,18] in diameter and are closely arranged [19] parallel to each other in 200–250 nm thick layers (or lamellae) [18] (Figure 3). The collagen fibrils themselves are weak light scatterers of light as their diameters are less than the wavelength of light with a refractive index close to that of the corneal ground substances [19]. Fibril packing is denser in the anterior two-thirds and axial cornea, in comparison to the peripheral cornea [20]. The lamellae are a hydrated matrix rich in proteoglycans, glycoproteins, salts and keratocytes [21]. Keratocytes that reside between the lamellae are responsible for the secretion of these components [22]. Within the central cornea there are approximately 200–400 lamellae [17,18,21] that span from limbus to limbus [23]. Lamellae organization and distribution is believed to control the corneal shape and curvature. 

The collagen fibrils are almost monomized in diameter (same shape and size) [1] across the majority of the cornea [16], but raises abruptly from approximately 4 nm from the centre to nearly 50 nm at the limbus [16]. Fibril diameter [16] and local interfibrillar spacing [1] does not alter with corneal depth, but has been shown to increase slightly with age [24]. The proteoglycans are thought to be responsible for the spatial distribution and organization of the collagen fibrils [3,24]. Electron microscopy and X-ray diffraction studies [25] indicate that the fibrils in adjacent stromal lamellae are predominantly orthogonal in arrangement [26]. It is the nanoscale organization of the stromal layer that is responsible for the corneas strength, clarity and its ability to refract light. It is also the principle reason why tissue engineered corneas have thus far failed to reach the clinic [1].

## 2. Diseases Linking to Scar Tissue Formation in the Cornea

Corneal diseases are vast and diverse with epidemiologies encompassing an array of infectious and inflammatory diseases. Corneal diseases are the second most common cause of vision loss and irreversible blindness to cataracts [27,28] globally affecting over 10 million patients [27,29] usually manifesting as a badly damaged ocular surface [28]. 180 million people worldwide are acutely visually disabled (although not classed as blind) discounting the further hundreds of millions affected by monocular visual loss [30]. The occurrence of corneal blindness is variable from country to country and between populations [30]. The prevalence is also accustomed to the availability and standards of eye care. For example, in some parts of Africa and Asia, corneal scarring is 20 times more likely than in industrialized countries. 

A great deal of research is focused upon diseases of the cornea as they are often responsible for changes in shape and thickness of the cornea [2] which may lead to permanent opacities which ultimately lower visual acuity [31]. Visual acuity is measured on a Snellen scale. The first number of the scale is the distance (in meters) away from the chart required to read it. This is usually 6 (*i.e*., 6 meters). As many rooms don’t have 6 meters available, a half-sized chart is used with the acuity expressed a ratio within a numerator of 6. The second line corresponds to the number of lines on the chart. 60 corresponds to the biggest letters, on the top line. The bottom line (*i.e*., smallest letters) corresponds to the number 6. Standard vision is defined as 6/6. The World Health Organization (WHO) definition of blindness is a visual acuity of 3/60 or less [30]. 

Historically, corneal blindness has been caused by disease including, although not exclusive to, trachoma, onchocerciasis, leprosy, opthalmia neonatotrum, xerophthalmia and opthalmia neonatorum. However, blindness due to ocular trauma, corneal ulceration and complications from using traditional medicines are becoming more commonplace as they often include dried plant materials crushed and dissolved into non-sterile aqueous medium, and may also include urine and saliva from animal or human sources, which present an excellent mechanism for pathogenic organisms to enter and spread into eyes that are already injured or infected [30]. A summary of corneal diseases specifically linked to scar tissue formation is provided in Table 1. 

**Table 1:** Corneal diseases linked to scar tissue formation; their epidemiology, causes and treatment.

Disease	Epidemiology	Causes	Treatment	Further information
**Fuchs dystrophy**	Deterioration of endothelial cells.			
	Loss in efficiency of pumping water from stroma.			
	Swelling and distortion of cornea.	Thought to be inherited, autosomal dominant trait [32].	Salt solutions such as sodium chloride drops or ointment are often prescribed to draw fluid from the cornea and reduce swelling.	Short-term success with transplantation, but long-term survival is a problem.
	Changes in the cornea’s curvature.	Gene mutation strongly suspected.	Contact lenses.	Cannot be cured.
	Hazing.		Hair dryer to dry out corneal blisters.	
	Tiny blisters on corneal surface.			
	Glare and light sensitivity.			
	Usually affects both eyes.			
**Ocular trauma and ulceration**	Unilateral vision loss.			
	Ulceration.	Mechanical trauma, debris entering the eye, chemical and thermal burns.		
	Corneal perforation Endophthalmitis.	Workplace activities, such as mining injuries, agriculture and warfare.	Corneal transplant.	Ocular traumas are becoming more prevalent causes of scarring and blindness.
	Phthisis.	Road accidents [33].	Antibiotic and antifungal treatments although visual outcome usually poor [30].	Worldwide, half a million people are blind as the result of trauma [30,33].
	Blindness.	Domestic accidents [33].		
	Hyphaemas [33].			
	Ruptured globes.			
**Opthalmia Neonatorum (conjunctivitis of the newborn)**	Bilateral scarring.	Infection caused by *Neisseria gonorrhoeae.*	Saline washes.	Blindness risk is reduced when opthalmia neonatorum is caused by less virulent pathogens such as Chlamydia trachomatis [30].
	Blindness.	Herpes simplex virus (HSV) can also cause childhood corneal blindness by causing opthalmia and xerophthalmia, although such infections are infrequent in infants.	Antibacterial eye ointments.	
	Affects both eyes.		Treatments with tetracyanite/erythromycin/silver nitrate ointments.	
**Stevens-Johnson Syndrome (SJS) also known as Erytheme multiform**	Subepithelial bullae.		Withdrawal of all potential causative drugs [35].	
	Scarring.	Drugs including sulphonamides, anticonvulsants, salicytes, NSAIDs, penicillin [34].	Intravenous fluid replacement.	<100 drugs associated with SJS [34].
	Keratinocyte apoptosis of the surrounding skin and epidermal necrolysis.	Infection, such as HSV.	Immunosupressive therapy.	Most severe cases referred to as toxic epidermal necrolysis (TEN)
	Erosion of mucous membranes [34].	Streptociocci, adenovirus and microplasma [35].	Corneal stem cell transplant.	Transplanted tissue often rejected.
	Scarring.		Corneal transplant.	
	Bilateral blindness [35].			
**Xerophthalmia (dry eye syndrome)**	Night blindness.	70% of cases are due to a vitamin A deficiency [30,36].	Artificial tears.	
	Xerosis.		Increase humidity of surroundings.	Xerophthalmia patients are predominantly infants or young children, with a peak age of approximately 2.5 years [36].
	Corneal perforation. Scarring.		Vitamin A supplementation.	All but disappeared in Western Europe [36].
	Irreversible blindness [36].			
**Trachoma**	Vascularization.	Bacterial infection trachoma caused by *Chlamydia trachomatis* [30].	Corneal transplant.	World’s leading cause of ocular morbidity and blindness [30].
	Ocular surface problems Entropion.	Infection can be transmitted from eye to eye via contaminated fingers, clothes, make-up and flies.	The disease is preventable via antibiotic treatment with azithromycin [37]; however more antibiotic treatment is still required to prevent further progression of the infection to corneal blindness in previously infected individuals [30].	
	Trichiasis.			
**Onchocerciasis (river blindness)**	Destructive chorioretinitis. Blinding keatitis	Caused by a parasite *Onchocerca volvulus* [30].	Invermectin kills the microfilaria (larval form) and sterilizes the adult worm to prevent spread in infected individuals.	Incidences of onchoceriasis have decreased since the introduction of invermectin in the 1980s.
	Acute corneal scarring.	Collagenase secretion, caused by the influx of inflammatory cells is believed to be responsible for the rapid destruction of the xerophthalmic cornea [36].		
	Vascularization.			
**Leprosy**	Blindness.	*Mycobacterium laprae* usually affects the anterior segment of the eye.	Multi drug therapy using dapsone, rifampicin and clofazamine.	Corneal complications caused by leprosy are a significant cause of corneal blindness globally affecting 250,000 people, predominantly in Africa and Southern India.
	Chronic uveitis.			
	Cataract formation. Exposure keratitis. Reoccurring corneal ulcers. Corneal scarring.			
	Vascularization.			

The majority of corneal diseases are the result of some form of disruption to the fibril arrangement in the stromal layer [3] and are a major cause of blindness worldwide. In areas of Africa in particular, over 90% of blindness is a result of corneal pathology, and the corneal scarring and vascularization is so severe that blindness is permanent. Unfortunately, many of the current treatments are not cost effective and can only alleviate or treat secondary symptoms of the disease and fail to cure the underlying cause. Prevention, in the cases of most diseases, would be more cost effective and successful in eliminating corneal blindness. However, more knowledge is still required to help us to understand the underpinning mechanisms that lead to permanent scarring in diseased and injured corneas. 

## 3. Mechanisms of Wound Healing in the Cornea

The ability of the cornea to remain transparent can become compromised when the cornea is exposed to trauma, infection, ulceration or chronic inflammation [38]. Much of the knowledge concerning corneal wound healing is derived from animal studies [39]. These are very valuable but anatomical differences such as the lack of a bowman’s layer in rabbit eyes mean that some caution should be exercised when extrapolating animal models to human clinical findings [39]. Corneal wound healing and the mechanisms involved are complex and include a cascade of cytokine mediated interactions between epithelial, stromal and endothelial cells, corneal nerves, lacrimal glands, tear film and cells of the immune system [40,41]. Activation of these mechanisms attracts immune cells which are responsible for eliminating debris and microbes that penetrate into the stroma [40]. However, it should be noted that most of the mechanisms that occur during wound healing are similar to those that occur during normal homeostasis [40]. In general, corneal wound repair occurs in 3 overlapping phases including re-epithlization and migration; cell proliferation and synthesis; and the remodeling of the underlying ECM [40,42] with many growth factors and cytokines stimulating one or more of these phases during repair [42]. Cell-cell cross-talk is vital to wound healing and occurs in a reciprocal manner, the signaling is not mutually exclusive and the action of one may depend or mediate the expression of the other [40]. The interaction of the cytokines leading to a network-like action is pivotal to the final outcome of wound healing [40]. However, the wound healing response and resulting tissue changes are dependent upon the size, depth [39,40] and inciting injury, with the severity of the response affecting the overall outcome [41]. Tear quality and integrity is also of importance as tear deficiencies can compromise the wound healing process [39]. The principal purpose of any wound healing response is to regain anatomical and functional capacity in the fastest and most perfect way. Elsewhere in the body, wound healing often ends in scar formation and vascularisation, where as one of the most vital aspects of corneal wound healing is the minimization of such results which would otherwise have serious visual consequences [39]. Healing of corneal tissue is slower than other connective tissues; possibly due to its avascularity [39]. 

The remodeling process of the injured stroma is vital to the resulting architecture of the tissue deposited following injury [41]. The synthesis, breakdown and cross-linking of collagen are all involved in the strengthening and stabilization of the wound and such processes can last for months to years. The remodeling process is able to alter the architecture of the initial repaired tissue so that it reverts to that of a non-injured state. Understanding these mechanisms and the factors involved may aid in the development of therapeutic agents which can speed up this process.

In mammals when a tissue is damaged a fibrotic response is usually activated which heals the tissue, but fails to restore full function [43]. However, in some instances such as during fetal wounding or some corneal wounding healing takes on a regenerative capacity whereby full function is restored. Corneal reaction to injury can be attributed to the architecture of the initial wound [44]. A theory of “activation” has been suggested by Fini *et al.* [43] where upon cells that undergo fibroblastic differentiation in response to injury will *repair* tissue; whereas cells that are able to proliferate in response to injury without activation will *regenerate* the damaged tissue. The subtle difference between the two could be the difference between an opaque tissue and a functioning transparent tissue. One of the most demanding challenges of corneal biology is to assist tissue repair via regeneration rather than fibrosis [45]. The cornea is a useful model for studying wound response and cell phenotype due to the unique homogenicity of the cell types that reside in the corneal stroma and remarkably organized tissue architecture that if disrupted has immediate and obvious effects [43,46]. 

### 3.1. Regeneration

There are a number of stem niches in ocular tissue that are vital to maintenance, repair and regeneration [1]; these include limbal stem cells (LSCs). LSCs are able to self-renew and generate daughter cells that can continue to differentiate until they are eventually shed [1]. *In vivo* the epithelium is regularly renewed from a population of relatively undifferentiated cells found in the basal layer of the corneal limbus. The limbus is the narrow ring of tissue located between the cornea and conjunctiva that ends anteriorly at the Bowman’s membrane. It supplies physical protection and nutrition to the stem cells. LSCs are normally slow-cycling, self-renewing cells that produce transient amplifying cells (TACs). TACs are capable of multiple rounds of division that does not usually occur during normal homeostasis [40]. Proliferation takes place which generates more TACs dependent upon the necessity, *i.e*., more proliferation occurs during regeneration (it can increase up to nine-fold) following a corneal wound. Usually, the average cycle time of a TAC is 60 h, but this can be shortened to 24 h upon stimulation [40]. The upregulation of stem cells and the increased proliferative capacity of TACs amplifies each cell division and reduces the need for stem cell proliferation whilst minimalizing the chance of introducing replicative DNA errors into the stem cell population which effectively provides a large number of terminally differentiated post mitotic cells [40] that move towards the centre of the cornea and upward from the basal corneal epithelial cell layer [1]. The final differentiated cells are eventually shed. The migratory epithelial cells are thought to move across the surface of the wound surface via a cyclical process involving philipodia and lamellipodia [39] that form temporary focal adhesions, whilst intracellular contractile mechanisms draw the cell forward. The focal adhesions are then cleaved enabling the process to be repeated in a cyclic fashion [39]. Epithelial repair is critical to the healing process [44]. Re-epithelialzation does not occur one layer at a time; there is a gradient of basal and suprabasal cells from opposite directions that meet in the centre [40].

The adjoining stromal layer is important to stem cell density, although the exact mechanism controlling this remains elusive. Complex intercommunications between the stem cells and their surrounding microenvironment are pivotal to this role [1]. The surrounding environment of LSCs and TACs differ and are principal to deciding cell fate in each region. Stromal regeneration, as opposed to repair, is dependent upon highly coordinated cellular interactions between epithelial and keratocyte cells [47]. Keratocytes originate from a population of neural crest cells. It has been postulated that some of the regenerative properties exhibited by these cells following corneal wounding could be attributed to their stem-like properties retained from their cell of origin [10]. An example of keratocyte regeneration is the healing of epithelial debridement wounds. In these cases, where the epithelium has been scraped away exposing the basement membrane, the keratocytes beneath the membrane undergo apoptosis. Mitosis of the adjacent cells occurs, which replace the cells, and no additional response occurs [10]. The initial cell apoptosis is thought to occur to prevent an inflammatory response occurring which may affect corneal transparency, which effectively protects the cornea from self-harm. However, the area and extent of cell death is variable and dependent upon the severity of the original injury and the species of the animal [10].

Preservation of the basement membrane has been attributed to the difference between regeneration and scarring [46,48]. The basement membrane is important in the regulation of epithelial-stromal interactions [46]. The presence of an intact basement membrane during wound healing is effective in hindering epithelial pro-fibrotic cytokines and growth factors [46], preventing their production whilst promoting re-epithilization. Thus the basement membrane provides effective anti-fibrotic protection to both the epithelium and stroma.

Reconstitution of transparency following injury is dependent upon the subtle mediation of biosynthetic activities of post-injury fibroblasts and matrix degradation attributed to proteolytic enzymes (enzymes that catalyze the splitting of proteins into smaller peptide fractions) [46]. It has also been suggested that regenerative healing is dependent upon the execution of 3 distinct phases: (i) fibroblast migration and secretion of proteases, ECM and growth factors; (ii) fibroblast differentiation into nonmotile, contractile myofibroblasts that secrete ECM proteins and remodel the ECM; (iii) wound closure and myofibroblast apoptosis [49]. It is the persistence of myofibroblasts that cause overproduction of ECM and excessive contraction [49] that lead to scarring and opacities. 

Investigations into experimentally injured adult rabbit corneas [50] have suggested that corneal regeneration recapitulates developmental stromal events regarding proteoglycan expression resembling that of early development. Corneal development has fascinated researchers for over 100 years [51]. It involves a precisely controlled sequence of events whereby the embryonic cornea undergoes key structural changes vital to its overall form and function. Embryonic chicks are predominantly used in the study of corneal development [51], however gene targeted deficient mice and zebra fish are also important model organisms in studying vertebrate corneal development [50]. 

Corneal embryogenesis, like regeneration, is dominated by important changes in structure and composition of the ECM. The collagen fibrils in the avian stroma are deposited in a two stage-process to form the corneal matrix. Although this two-stage process is not so obvious in non-avian corneas, differences in the outer and inner corneal development are still apparent in human corneal development. The corneal epithelium inhabits a large section of the corneal thickness (69%) during early development, consisting of undifferentiated multi-stratified cells lacking in the usual layered organization [50]. Initially, the epithelial cells secrete a primary matrix which is thought to act as a template for the following biosynthesis of the secondary stroma by the neural crest cells which later become keratocytes [51]. The epithelium increases in thickness (80% overall thickness) as it becomes more developed and organized. The cells arrange into 3–4 distinct layers [50] which eventually flatten out thus reducing epithelium thickness. The primary evidence of corneal ECM is a very thin layer [50] of fine collagen fibrils, known as the “primary stroma” which is a product of ectodermal cells (one of the 3 primary cell germ layers) [51]. A two-wave mesenchymal cell migration occurs from the periphery into the primary stroma. Most of the early migrating cells will form the endothelium; the secondary cells will become stromal fibroblasts. The invading cells cause stromal swelling and an increase in thickness. The corresponding widespread invasion of fibrotic mesenchymal cells, destined to become keratocytes, begin to populate the stroma and synthesize much of the secondary stroma by depositing collagen fibrils as coarse lamellae [51]. It is thought that the migrating cells use the primary stroma as a template; however the exact mechanism is unclear. The stroma increases in thickness before undergoing a marked compaction process as water is lost, concentrating the matrix components and at the same time increasing transparency. The cornea is thicker in the middle stroma compared to the anterior stroma [50]. Proteoglycans are pivotal to corneal morphogenesis; they interact with collagen and control the size, diameter and arrangement of the fibrils whilst helping to determine the hydrophobicity and swelling properties of the matrix [51]. They also aid in cell migration and cell differentiation. Initially there are only a small number of proteoglycans which aggregate below the epithelium and above the endothelium, significantly increasing in size and number as the cornea develops [50]. 

During early corneal development the collagen fibrils are already in an orthogonal order. The lamellae are progressively deposited and rotate in sequence beneath the epithelium. The direction and rotation of the lamellae is a self-assembly process whereby the matrix itself dictates the evolving architecture [51]. Influencing orientation factors are largely unknown, however may be due to biomechanical stresses in the stroma caused by intraocular pressure and direct interactions between matrix components [51]. 

Fibrillogenesis occurs within small surface recesses of keratocytes with small bundles between 5 and 12 fibrils. The smaller bundles combine into larger bundles, before eventually becoming lamellae [51]. The number and orientation of the lamellae become more established with increasing development and successive synthesis and addition of more collagen and ECM components. Dehydration of the cornea becomes apparent as a thinning of the secondary stroma followed by structural reorganization of cells and ECM [51]. The endothelium contributes greatly to the dehydration process although the exact role is not known. The endothelium itself is initially very thick (23% corneal thickness), but decreases in thickness over time [50]. Small stromal proteoglycans, such as keratocan sulfate may mediate the fibrillar organization on a localized level via interactions with collagen and each other, rather than having a global matrix compaction effect [51]. 

### 3.2. Scar Formation

Although the cornea is exceptionally resistant to immune response and stimuli in comparison to other tissues in the body [43], scaring of the cornea can occur for many reasons. Debris and/or chemicals entering the eye, infection, inflammation and diseases of the cornea can all lead to permanent scarring. Scar tissue formation often changes the optical properties of the cornea, thus altering sight. Injuries to the cornea manifest as changes in cell phenotype particularly in the stromal layer. The disruption of the Bowman’s layer and Descemets’s membrane are also both key steps in initiating tissue remodeling [46]. A fibrotic response usually involves rapid contraction and closing of the wound space by activated keratocytes (fibroblasts) followed by scarring. However, keratocyte activity does not manifest until the corneal surface has been fully re-epithelialized [39]. In the case of fibrotic response in corneal wound healing opaque scar tissue will ultimately interfere with vision and the strength of the corneal scar tissue will never match that of uninjured tissue [39]. The primary stages of wound healing involve removal of the injured tissue, followed by cell proliferation and migration to the wound site. Here repair and replacement occurs before the wound healing procedure has ended [52].

During scarring a change in collagen or proteoglycan structure alters the usually highly organized lattice structure, thus decreasing the optical properties of the cornea. Corneal scar tissue is hazier, lass elastic, with lower mechanical properties than a normal, healthy adult cornea [47]. The structural origin for the haziness of scars is debated between researchers. Increased cellularity of the scar [38], vacuolation (an increased number of vacuoles present), or light scattering occurring due to a modified collagen fibril arrangement are all plausible explanations. In electron microscope studies on scarring [47] new scars have been shown to contain vacuoles (vesicles found within the cytoplasm of cells) with collagen fibrils with a normal average diameter, but a larger range of diameters. Transparency can improve over time as the spacing between the fibrils nears normal, although vacuoles remain. Even after prolonged periods of healing (up to 18 months), original collagen structure and cross linking is not restored, thus scar remodeling is never perfect and remains incomplete, *i.e*., it never returns to its pre-injured state. 

Damage to the corneal endothelium has also been well documented, as endothelial integrity is pivotal to the maintenance of corneal transparency [39]. The endothelial cells are often disrupted during corneal, trauma, disease and surgeries which leads to stromal dehydration and hazing [42]. Additionally, over hydration of the endothelium can also cause corneal opacity. This is due to the loss of the tight junctions and integrity of the epithelium during wounding, which consequently affects cell membrane permeability leaving the cornea more vulnerable to infections by microbes [40]. The invasion of microbes disrupts cytokine-mediated control of healing which decreases endothelial fluid transport, thus increasing stromal hydration [40]. As many as 80% of endothelial cells can be lost before decomposition occurs [39]. Endothelial cells in humans and primates have minimal or no capacity to proliferate and thus endothelial wound healing is dependent upon the enlargement and migration of the surrounding cells to cover the wound site [53]. Additionally, endothelial cells *in vitro* have limited proliferative capacity [42] and so the use of growth factors may have a key role in regulation and stimulation of the healing endothelial layer. 

In some contexts scar formation represents fetal corneal synthesis in that the fibrils secreted in the initial stages of a scar are comparable to the diameter of the parallel bundles of fibrils secreted during embryogenesis [47,54]. Additionally, activated keratocytes express fetal surface antigens for up to 6 weeks following injury. However, differences are apparent with respect to the reduction in the ratio of interfibrillar type VI collagen to type I collagen in scar tissue compared to the developing cornea, and lack of the original organized template in fetal tissue.

## 4. Effectors to Control the Outcome of Corneal Wound Healing

### 4.1. Phenotype Differentiation in the Corneal Stroma

**Figure 4:**
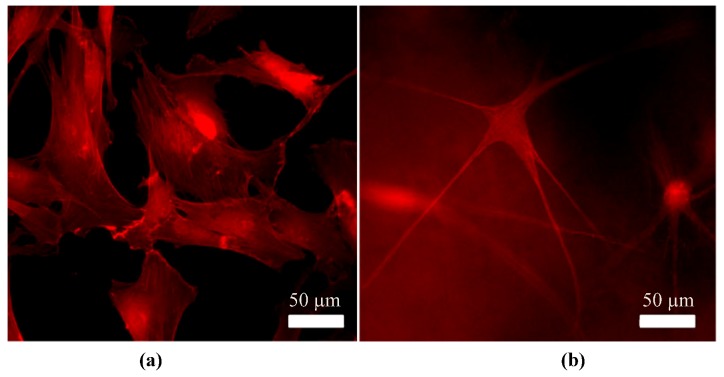
F-actin stained fluorescent images showing differences in morphology of (**a**) a fusiform fibroblast compared to (**b**) a more dendritic shaped keratocyte.

Quiescent keratocytes inhibit the stromal layer in a healthy cornea *in vivo* [43,55,56,57,58]. When viewed in cross section they appear as sparsely populated flattened cells located between the collagen lamellae. However, when viewed *en face* they appear as broad cells that form an elaborate network [43] interconnected by long dendritic processes [10] (Figure 4b**)** joined by gap junctions [48]. Keratocytes are responsible for the synthesis of the stromal ECM which is vital to maintaining transparency. The main function of the keratocyte is to maintain the balance of stromal substances by synthesizing new collagens and proteoglycans, whilst secreting collagenase amongst other enzymes to degrade old stromal matrix [58]. Keratocytes contain highly expressed proteins known as crystallins, which contribute to the transparent nature of the cornea. 

During wound healing, an epithelial slide is initiated at the wound edge. This is independent of cell division and cell migration is achieved via a migration of slow cycling stem cells situated in the basal layer of the limbus [47]. The sliding cells release plasminogen activators (a precursor to plasmin) that cleave transient macromolecular focal contacts released as the epithelial cells migrate. The epithelial layer thins over protruding areas of the wound and thickens over the wound area, effectively producing an even, smooth corneal surface [47]. The epithelial cells co-ordinate with the keratocytes in the stromal layer below via cytokine release mechanisms, which aid and stimulate migration and proliferation. The cytokine release attracts inflammatory cells (such as macrophages and T-cells) into the stroma, resulting in an inflammatory response [45]. Keratocyte migration back into the original wound site only occurs once the wound surface has been re-epithialized. 

This inflammatory response initiates phenotypical changes to the keratocyte. Previous *in vivo* studies on rats [43], cats [59] and rabbits [43,60] have enabled the changes of a keratocyte into an activated repair fibroblast to be observed [43]. Keratocytes in close proximity to the wound edge undergo apoptosis and disappear within a few hours, leaving an acellular area. This activates the keratocytes flanking the acellular area within 6–24 hrs after the initial injury. Ultrastructural and biochemical studies demonstrate that keratocytes are markedly different from fibroblasts (Figure 4b) with regards to intracellular distribution of filamentous actin and form stress fibers characteristic of myofibroblast-like cells [61]. These changes allow usually quiescent cells to migrate to the injured area and proliferate. This process can last up to 7 days. Corneal fibroblasts are greater in size than keratocytes with a fusiform shape. They secrete higher levels of ECM proteins and enzymes [62,63] and so are able to reconstruct and repair the injured tissue. The differences in composition of ECM synthesized by corneal fibroblasts in comparison to keratocytes may account for the opacity of the resulting repair tissue [43], as the resulting scar tissue has a different composition compared to healthy tissue. Epithelial cells are gradually moved upwards and their remaining attachments are combined into the remaining scar. Keratocyte activity reverts to normal usually after 3 months [47], although hypercellularity (an abnormally large number of cells) can remain for years.

During wound closure a subpopulation of corneal fibroblasts appears: the myofibroblast. Myofibroblasts are greater in size, less mobile and exert greater contractile forces on a tissue in comparison to fibroblasts [64]. Myofibroblasts are fundamental to wound contraction and scarring [49,65]. They are characterized by the presence of actin fibers, including α-smooth muscle actin (α-SMA) [59,64] which helps to convey contractile properties of the cell [43,59]. Optical transparency may return with time, but even after 6 months the cells in the scar are not normal keratocytes, although they may share many similar characteristics [43]. Corneal fibroblasts and myofibroblasts have been identified *in vivo* many weeks after the injury has occurred [8,66]. It is still not fully explained if the fibroblasts and myofibroblasts ever fully revert back to the uninjured keratocyte phenotype both morphologically and biochemically. It has been hypothesized [57] that corneal fibroblasts can partially return to the original keratocyte phenotype but some fibroblastic characteristics are retained [57,67]. It has been suggested that myofibroblastic differentiation is not activated by stromal injury alone and it is in fact a consequence of both stromal and epithelial injury [65]. The precise involvement of the corneal epithelium in corneal stromal wound healing is not known. However, deviations of its barrier function render it permeable to cytokines and growth factors from the tear film. These may then penetrate into the stromal layer, thus causing activation of the residing keratocytes [48]. 

In summary, it is possible to induce keratocytes isolated from a natural cornea to differentiate into 3 (possibly more) different phenotypically different cell types; keratocytes, fibroblast and myofibroblasts [55].

### 4.2. Growth Factors

In a healthy, uninjured cornea cell generation and loss, and the quantities of growth factors that stimulate and inhibit, are balanced. Injured cells generate cytokines- powerful peptide or glycoprotein regulatory factors that serve as autocrine or paracrine agents to control cell communication, migration, proliferation, protein synthesis [47] inflammation, differentiation and angiogenesis [52]. Cytokines regulate both epithelial and stromal wound healing responses [1]. The cytokines are thought to originate from the lacrimal gland, conjunctiva and conjunctival vessel that are secreted into the tear film during would healing with differing concentrations measured in accordance to different corneal pathologies [52]. 

Wound healing is regulated by a vast assortment of growth factors, including but not exclusive to platelet derived growth factor (PDGF), epidermal growth factor (EGF), transforming growth factor beta (TGF-β), basic fibroblast growth factor (FGF, also known as bFGF and FGF-2), the interleukins (Il-1, Il-6, Il-8), tumor necrosis factor alpha (TNF-α) [47], insulin-like growth factor (IGF) [68], keratinocyte growth factor (KGF), hepatocyte growth factor (HGF) and secreted protein, acidic and rich in cystine (SPARC) [65]. *In vivo* all these growth factors differentiately co-ordinate keratocyte proliferation, cytoskeletal organization and ECM synthesis [68] via a complex cascade mechanisms [41]. Growth factors are able to act on corneal tissues through autocrine (self-acting, the growth factor acts upon the cell that produces it), paracrine (growth factor acts upon cells in the vicinity of the emitting cell) or juxtacrine (affecting both emitting cells and cells in the vicinity) fashions [69,70] and the presence of multiple growth factors have varying responses dependent upon their relative concentrations [70]. For example, KGF and HGF act via paracrine mechanisms where as TGF-β mediates as a paracrine system; PDGF can act via juxtacrine mechanisms [69]. The effects of growth factors on a cell type can vary dependent upon ECM environment [70]. The binding of growth factors to ECM leads to sequestration or controlled release that may regulate or enhance the growth factor effect [70]. Upon binding, integrin signaling, shape and cytskeletal tension which control cell cycle progression initiated by growth factors are changed [70]. Cells stimulated by growth factors cause them to produce or alter ECM and promote migration via enhanced integrin expression, affinity or cytoskeletal interactions [70].

Epithelial cells secrete type I cytokines including TGF-β, Il-1, and PDGF [1]. TGF-β and PDGF in particular are powerful chemotactic agents and mitogens (stimulate mitosis) for fibroblasts and can initiate wound contraction [17,65]. TGF-β is arguably the most documented growth factor associated with corneal wound healing in literature and recent investigations into molecular therapy have been focused upon the inhibition of myofibroblast differentiation via the targeting of TGF-β isoforms [41]. The TGF family can be divided into two subfamilies, TGF-α and TGF-β [69]. TGF-α is similar to EGF in that it stimulates epithelial proliferation and has a common receptor; whereas TGF-β is very different and is secreted by almost all nucleated cells [69]. *In vivo*, the TGF-β superfamily are structurally related [71,72], mulitifunctional growth factors principally related to fibrosis, myofibroblastic differentiation [49,65,68,73,74], embryonic development, increased granulation [71] and contraction in many cell types; and loss of transparency specifically in corneal tissue. Three isoforms of TGF-β have been identified in mammals, TGF-β1, -β2 and TGF-β3 [40,41,69,70,71,73,75]. TGF-β1 and TGF-β2 have been localized in both the corneal epithelium and stroma and are both components of tear fluid [72]. mRNA of the TGF-β3 has been isolated from whole rat corneas in low levels, although its exact tissue location remains unclear and it has yet to be isolated and detected in non pathological corneas [72]. The receptors for TGF-β1 and TGF-β2 have been located in all three corneal cellular levels, being most prominent in the basal layer of the corneal epithelial cells [72]. The receptor for TGF-β3 has been located in the epithelium and endothelium *in vivo*, but is not present in native keratocytes [72].

TGF-β1 is the most commonly documented isoform that induces fibroblastic and myofibroblastic differentiation, although the TGF-β2 and TGF-β3 isoforms have been shown to elicit a similar transformation [73] with regards to stimulating cell stratification and matrix component production (such as collagen type I deposition) [71]. However, many studies have also demonstrated that despite the fact that the three isoforms share binding sites and are capable of activating the same effect of receptors; the overall effects are markedly different [71]. TGF-β2 has been established as the primary mediator of fibrosis and scarring as it is believed to play a role in the development of excessive ECM build up [73] and along with TGF-β1 increases fibrotic gene marker expression [71] such as α-SMA and collagen type III. Both TGF-β1 and -β2 are very potent growth factors that are capable of blocking epithelial, lymphocyte and hepatocyte cell proliferation promoted by KGF, EGF and HGF [69,70], whilst stimulating the proliferation of mesenchymal originating cells such as fibroblasts. Interestingly, the TGF-β3 isoform has been shown to act as an inhibitor of fibrosis and scar development [71], and has been shown to promote scar-free healing. TGF-β is auto-inductive for fibroblasts (secrete more and more) therefore an additional process is required to stop the stromal repair mechanism once the wound is closed [47]. 

PDGF exists as a dimeric glycoprotein comprised of two A (AA) and two B (BB) chains, or a combination of the two (AB) [69]. PDGF can facilitate TGF-β myofibroblast differentiation via an autocrine feedback loop [68]. PDGF-BB stimulates keratocyte proliferation, upregulation of the secretion of normal stromal ECM and migration of both keratocytes and fibroblasts [68,76]. Thus PDGF-BB may regulate stromal migration and proliferation following injury without initiating the production of fibrotic tissue. PDGF-AB on the other hand has been shown to differentiate keratocytes to fibroblasts, evident via the development of stress fibers, local adhesions and contraction in 3D matrices [68]. Similarly, PDGF-BB is able to significantly increase the growth of endothelial cells *in vivo* where as PDGF-AA does not [69]. However, neither TGF-β, Il-1, nor PDGF will exert their proliferation, chemotaxis or differentiation effects on stromal cells under normal homeostatic conditions [46].

Il-1 and soluble Fas ligand are cytokines released by epithelial cells in response to injury [77] and have been associated to the upregulation of HGF [41] and KGF [40], and acting in synergy with EGF to accelerate epithelial wound closure [40]. Il-1α is a master modulator of the wound healing cascade [41] and has been linked to fibroblast apoptosis via the induction of Fas ligand mRNA and protein production that results in autocrine suicide [77]. TNF-α has also been linked to keratocyte apopotosis [41]. Il-1 released from the epithelium at the apex of the epithelial cells following apoptosis. It is also present in the tears in conditions associated with ocular surface injury [41]. Unwounded keratocytes to not produce Il-1α, but the Il-1α autocrine loop is initiated in corneal fibroblasts in response to Il-1 stimulation [77]. Keratocytes only produce Il-1 when exposed to the cytokine following epithelial injury as it cannot pass into the stromal layer as the epithelial barrier functions blocks it. Keratocytes exposed to Il-1 enter a self-perpetuating autocrine loop [41] that controls the production of HGF and KGF by keratocytes which effectively regulates epithelial healing and keratocyte proliferation and apoptosis [69,77] via the Fas-Fas ligand system. Il-1 also initiates the synthesis of collagenases, MMPs and other enzymes by keratocytes which are involved in collagen remodeling during healing. Il-1 and PDGF are usually only secreted by epithelial cells under physiological conditions, where as their corresponding receptors are expressed by stromal cells [46], demonstrating the need for cross-talk between cell types. 

Type II cytokines moderate both epithelial and fibroblast cells and include IGF-1 and FGF. IGF stimulates keratocyte proliferation and migration [69] whilst maintaining cell morphology, cytoskeltal organization, and keratocan sulfate synthesis. It has also been shown to enhance the effects of EGF on endothelial cells [39] and exists as a component of tears [69]. EGF mRNA has been detected in all corneal cell types [53], and the protein is present in endothelail and epithelial cells, but to a less extent in stromal cells [70]. EGF exerts its effects on cells via paracrine, juxtacrine and autocrine mechanisms [70]. EGF has been associated with corneal vascularization in some studies, whilst having no apparent effects in others [70]. Thus angiogensis via EGF may be linked to its combined presence with other growth factors, FGF in particular [70], during wound healing. 

Fibroblasts produce type III cytokines including KGF and HGF which are important signaling molecules in regulating epithelial-mesenchymal interactions [52]. KGF and HGF (also known as scatter factor [70,78]) both stimulate epithelial cell proliferation in a paracrine fashion [40] by activating their corresponding receptors [46]. KGF (a member of the FGF family [69]) stimulates growth of limbal and conjunctival epithelial cells where as HGF is involved in the proliferation and control of peripheral epithelial cells, with the dominant factor dependent upon the wound site [40]; EGF has similar effects and is a known potent mitogen of epithelial cells [70]. It is thought that EGF acts as a basic facilitator for epithelial proliferation where as KGF and HGF are up-regulated dependent upon the amount of damage [40]. HGF production is increased in the lacrimal gland following epithelial injury, and thus this may be the primary source of HGF during early wound healing until the fibroblasts/myofibroblasts repopulate stroma [41]. HGF also promotes epithelial motility, aiding in the spatial arrangement of the cells [78] and prevents epithelial terminal differentiation [46]. The mitogenic activity of KGF is restricted to epithelial cells *in vivo* where it acts as a paracrine effector [69]. KGF is produced by keratocytes and acts on the epithelial cells by binding to KGF-receptors [69]. 

During wound healing, the initial removal of the injured tissue is controlled by plasminogen-activator/plasmin systems and matrix metalloproteinases (MMPs) [52] which degrade the components of the ECM. Plasmin cleaves the proteins of the ECM and initiates dominant enzymes such as pro-collagenase and TGF-β. MMPs are zinc-dependent proteinases whose substrates contain several elements of the ECM and basement membrane [44] that participate in ECM remodeling and angiogenesis (the development of new blood vessels) [52]. MMPs are deposited in a dormant form by the resident cells and invading inflammatory cells [40,41] and are organized via tissue inhibitors of metalloproteinases (TIMPs). They are vital in embryogenesis, development, and regulate wound healing via cell migration and contraction [44]. In a healthy cornea, only low amounts of MMP-2 are detected as a proenzyme. However, following injury and cytokine release, many MMPs are upregulated. MMP-1 (interstitial collagenase), -2 (gelatinase A), -3 (stromelysin-1) and -9 have all been shown to have involvement in epithelization and stromal remodeling [44]. The precise mechanism of MMP action remains elusive; however MMP-1 is known to be vital for epithelial migration *in vitro* [44]. MMP-9 has been associated with basement membrane deterioration and MMP-2 and -3 are thought to manage lasting stromal remodeling and membrane synthesis [44].

Wound models on rabbit eyes were performed by Mulhollend *et al.* [44] in order to understand the healing response following photorefractive keratectomy (PRK) or laser *in situ* keratomileusis (LASIK) procedures. Following wounding MMP-2 and -9 were primarily confined to behind the leading edge of the migrating epithelium. This demonstrated that these enzymes may be involved in stromal remodeling and/or early basement membrane construction. MMP-2 involvement in stromal remodeling is thought to be separate from epithelial repair [44]. MMP-9 is likely to act as a factor in stromal repair as it can prompt TGF-β (a dominant modulator of scarring). In studies on knock-out mice MMP-9 has been shown to increase re-epithelization and has been associated with basement membrane formation [44]. MMP-7 can prevent secondary neovasculation of the wound. MMP-1 activation is thought to be crucial to epithelial cell migration and wound closure. MMP-2, -9 and -3 have all been connected to keratocyte migration and fibroblast activation at the wound site, construction of basement membrane and stromal remodeling [44]. By developing and prompting specific MMP inhibitor the wound healing process can be separated and specific stromal responses can be indentified and interrupted, thus preventing a fibrotic response whilst conserving epithelialization. 

### 4.3. Matrix Components

The majority of the corneal stroma is comprised of Collagen I, V, VI and XII, along with dermatan sulfate proteoglycan decorin and keratan sulfate proteoglycans (KSPGs) lumican, keratocan and osteogycin [19,79] which form the ground substances of the stroma. Keratan sulfate comprises of approximately 50% of all the stromal glycoaminoglycans (GAGs) present in the stroma [19]. Keratocytes are the major cell type in a healthy cornea. As of yet there is no single molecular marker for keratocytes defined although they do express lumican and keratocan to develop and maintain collagen interfibrilliar spacing and fibril diameter [79]. 

Keratocan is a cornea specific protein only expressed in healthy corneas and keratocytes [79]. *In vivo*, following initial injury keratocan expression is lost. It may return during and following the healing process, albeit in significantly lower levels than in an uninjured cornea [79]. Similarly, *in vitro* keratocan expression is lost when stromal cells are cultured in the presence of serum [67,79].

Lumican belongs to a small leucine-rich proteoglycan family and is a major KSPG [80]. The non-glycanated lumican core protein is distributed extensively in numerous interstitial connective tissues including the sclera, aorta, cartilage and liver [80]. However, in the cornea it is found in a glycanated form, meaning keratan sulfate GAGs (KS-GAGs) have bound to the central protein. This binding is thought to be involved in the control of fibril diameter, with the extended KS-GAG side chains controlling fibril spacing and corneal hydration required for tissue integrity and transparency. In studies on lumican knock-out mice, abnormally large collagen fibrils and disorganized interfibrillar spacing occurs [80]. This confirms that the key role of lumican is concerned with maintenance of fibril architecture via fibril assembly arrangement and balancing the KS-GAG levels required for transparency. It may also act as a regulatory molecule for cellular functions including proliferation, migration and prevention of apoptosis in injured epithelium, and modulation of keratocan and aldehyedehydrogenase (ALDH, an important modulator of corneal metabolism) expression via keratocytes [65,81]. 

Decorin is a small leucine rich protein that plays a key role in many cellular tissues in a variety of tissues including the cornea [81]. It is the predominant dermatan sulfate proteoglycan in the cornea [82] but is also found in the majority of connective tissues. Decorin is expressed in both healthy and injured corneas [82] where it interacts with growth factors such as EGF and TGF-β to mediate processes such as fibrillogenesis [83], ECM metabolism, cell-cycle progression [81] and remodeling following injury. Decorin expression increases significantly in response to injury which has led to it often been associated as a marker for corneal fibrosis and scarring [82]. Apparently, decorin is a natural inhibitor of TGF-β, a growth factor strongly affiliated with myofibroblast differentiation, that results in corneal haze and scarring. It is expected that decorin is able to interact with collagen, thrombospondin and inhibit TGF-β expression, thus preventing myofibroblastic differentiation, and effectively reducing the fibrotic response [83]. Recently, decorin has been investigated as a potential gene therapy to treat corneal haze *in vivo* [83]. 

Scar tissue alters the constituents of the original ground substance of the cornea. The proteoglycans, type III and IV collagen filaments and various other proteins and glycoproteins constitute to the ground substance between collagen fibrils. They are vital in maintaining tissue homeostasis with regards to regulating water content, collagen fibril diameter and spacing and thus ultimately transparency. Staining of the proteoglycans [47] has been used to demonstrate changes in the ground substances as a result of scarring. For example, in a new scar tissue, the presence of fibrotic markers such as type III collagen, fibronectin and α-SMA are increased [75]; enlarged proteoglycan [47,54] fibrils and reduced keratocan sulfate expression are observed [47]. These abnormalities have been attributed to increased water content and the appearance of vacuoles, which contribute to corneal haziness [47] due to disruption of fibril organization [54]. However, differences in wound type, *i.e*., inflammation, necrosis, ulceration and scarring cause different wound healing responses due to differing ultrastructural changes [54]. This has been attributed to the differences in the cytokines and growth factors expressed which vary with wound type [79]. However, the healing process can usually be identified by global inflammation and swelling [80]. 

Healing corneas share many characteristics of the developing cornea in that they both appear to have unusually thick collagen fibrils and irregular spacing and primarily synthesize collagen types I and V [54]. Heavily opaque corneal scarring has also demonstrated the phenomenon of abnormally large fibril diameters. The increase in collagen fibril diameter in response to injury prevents normal swelling in the subsequent scar tissue from developing, causing disruption of corneal homeostasis [54]. The enlargement of the proteoglycans usually responsible for regulation of fibril spacing may physically increase water uptake. Proteoglycans are hydrophilic molecules and their presence may increase spacing between the fibrils making them appear larger [54]. However, studies performed on bovine corneas [54] demonstrate that intermolecular spacing between collagen fibrils is similar in both injured and uninjured corneas. Thus the increase in fibril diameter is not necessarily caused by the swelling usually observed during healing that may push the collagen molecules apart. The increased fibril diameters may be due to a fusion of adjacent collagen fibrils, caused by a deficit of proteoglycans in the newly synthesized tissue [54]. Defects in the mechanism that usually controls fibril diameter, mediated via type V collagen synthesis may also play a role. 

In addition, type III collagen is also synthesized in both normal (albeit in low levels) and developing corneas [84,85]. Type III collagen is one of the earliest collagens to be laid down during growth and regeneration [84]. Type III collagen is associated with the normal adult descemets membrane and the corneal tissue of most animal species are able to synthesize type III collagen, although the age and physiological state of the cells responsible for synthesis and deposition are debated in literature [84]. Expression of type III collagen is initially significantly increased [75] in healing corneas, thought to be synthesized and deposited via endothelial derived fibroblasts; however it is easily broken down during tissue remodeling [84]. It has been speculated that the intermolecular disulphide cross-links within the molecule provide mechanical stability during the initial formation of scar tissue [84]. Most corneal fibroblasts express collagen types I and III simultaneously [85]. The conditions whereby fibroblasts are able to synthesize type II collagen corresponds with the conditions whereby they lose the ability to synthesis keratan sulfate. It is thought that the exposure to proteolytic enzymes and fibroblast differentiation affects the initiating type III collagen synthesis [85]. 

### 4.4. Mechanical Stress

The tensile mechanical strength of the cornea is vital to its function and the mechanical properties are essential for linking morphology to mechanical behavior [23,86,87,88] as fibroblast interactions within the collagen matrix are decided partially by the cell-matrix tension state [76]. It is the biomechanical and wound healing capacity of the cornea that determines the reproducibility and stability of many refractive surgery techniques [23]. The overall biomechanical properties of the cornea are complex due to the anisotropic nature of the tissue, *i.e*., different mechanical properties in different directions [1]. The anatomical and regenerative characteristics of the cornea are vital to its function as a strong, transparent structure able to protect against intraocular injury [23] without rupture [1]. The highly organized and ordered collagen fibril network accounts for the corneas mechanical strength [20,89] and load bearing element within the tissue [89]. The interweaving collagen bundles between adjacent lamellae supply vital structural foundations for sheer (sliding) resistance and transference of tensile loads among lamellae [23]. The load bearing capacity is concentrated in two main directions; the temporal-nasal (side-to side) and superior-inferior directions (up-down) [89]. Collagen fibrils are only found in the stroma and Bowman’s layer; hence it is these layers that are vital in provision of the cornea’s tensile strength [23]. Stripping of the epithelium, although has biological ramifications, has little to no effect on the anterior corneal curvature [23], thus attributes very little to the corneas overall tensile strength. The Descemets’s membrane has a relatively low stiffness and so accommodates for fluctuations in intraocular pressure which may attribute to the absence of stromal stresses experienced by the adjoining endothelium [23]. The Bowman’s layer is an 8–12 µm layer that consists of randomly orientated collagen fibrils [90]. Extensiometry testing whereupon the Bowman’s layer was removed resulted in negligible mechanical changes of the cornea, thus the mechanical properties of the cornea are stromal layer dominant [23].

The cornea acts as a scaffold for the refractive surface of the eye, thus every mechanical or biological reaction to disease and/or injury have an impact on visual acuity [23]. For example, when the cornea becomes dehydrated, the stress is distributed largely to the posterior layers or regularly over the whole cornea [20]. In a healthy or edematous (swollen) cornea, the anterior lamellae assume nearly all of the strain [20]. Stress within the tissues is connected with intraocular pressure. Keratorefactive treatments and corneal disease may alter the biomechanical properties directly or indirectly [20]. 

The algorithms and models that many refractive ablation techniques are dependent on generally assume that the cornea is biologically and biomechanically passive, thus all patients outcomes should be predictable and alike [23]. However, this is evidently not the case as the cornea often has a fibrotic response to incisional and ablative procedures. Biomechanical deviations become apparent with changes in corneal shape, shape instability, enhanced sensitivity, changes in hydration, hypoxia (oxygen deficiency) and injury as a consequence [23]. During scarring, deposition of abnormal tissue reduces transparency whilst disorganized loss of the remaining tissue causes a loss of tensile strength [52]. *In vivo*, large fluctuations in mechanical phenotype caused by ECM tension can be generated via lacerating injury, PRK and refractive surgery techniques [68]. Additionally cell matrix tension state is controlled by collagen density, matrix restraint and growth factor environment [76]. 

During healing, the initial matrix is less dense, disorganized and more flexible than uninjured tissue. ECM composition, stress and stiffness can all alter the cell phenotype both *in vivo* and *in vitro*. For example, increasing ECM stiffness *in vitro* is known to up regulate myofiroblastic transformation, mimicking what occurs *in vivo* as myofibroblasts develop towards the end of the wound healing process when cell density is high and the wound environment is stiffer [68]. The increased cell-matrix tension causes fibroblasts to display stress fibers, focal adhesions and activate focal adhesion signaling via phosphorylation of focal adhesion kinase [68,76]. This response is absent when cell-matrix tension is low, *i.e*., in a healthy cornea as from a mechanical point of view quiescent non-activated keratocytes do not generate stress fibers or vast contractile forces [17]. The dynamic mechanical feedback existing between cells and the surrounding matrices in three dimensional (3D) *in vitro* cultures occurs due to time-dependent ECM remodeling, and may facilitate patterning during embryonic development as well as during wound healing [17]. 

## 5. Refractive Surgery and Scar Tissue

With the trend occurring that most individuals will at some stage in their life go on to develop some form of refractive error, ophthalmic surgery needs to develop and progress in order to provide sufficient treatment for preserving or restoring vision. Jose Barraque first recognized that small changes in the curvature of the cornea can dramatically affect its refractive ability, and in 1976 introduced the first lamellar refractive surgery techniques [91]. Photoablastion of corneal tissue was reported by Trokel *et al.* in 1983 [92] and showed evidence of changes to the refractive power of damaged corneas. Since then, variations of refractive surgery techniques have come into practice, with the common aim to alter and improve the refractive power of the cornea. Photoreactive keractectomy (PRK), Laser subepithelial keratomileus (LASEK) and Laser *in situ* keratomileus (LASIK) are all types of refractive surgery to the surface of the cornea and involve removal of corneal tissue. Differences in procedures occur with the subsequent treatment of the epithelium [91]. Laser thermal keratoplasty (LTK) and conductive keratoplasty (CK) include heat generation to shrink the corneal stromal and central corneal steepening [91]. Radial keratotomy can be used to flatten the cornea via number of radial incisions. 

### 5.1. Refractive Surgery Techniques

Thermalkeratoplasty (LTK) and conductive keratoplasty (CK) heat the cornea to a critical temperature of 58–76 °C inducing collagen shrinkage, thus altering the corneal curvature to make it steeper [20]. The uses of such techniques are reserved for treatments of the mid periphery cornea. LTK is a non-contact treatment whereby a holmium laser is used to heat the corneal collagen. CK is a contact treatment involving the use of a radiofrequency diathermy probe inserted directly into the cornea [20]. Both techniques have problems associated with local necrosis if the temperature is too high and if the heat source is not consistent or uniformly administered, variable astigmatism (unequal corneal curvature) arises [20]. 

Radial keratotomy (RK) is an incisional technique used to treat myopia and hyperopia. However, the technique is often superseded by excimer laser treatments. It continues to have a role in the treatment of primary and residual astigmatism following cataract keratorefractive surgery and photorefractive keractectomy (PRK) [20]. RK differs from surface ablation and LASIK procedures in that it excludes the removal of tissue from the central cornea. The procedure includes making tiny cuts in the cornea, which flattens it, reducing the curvature of the cornea [91] and decreasing the refractive power [20]. Drawbacks affiliated with RK include daily fluctuations in vision and excessive flattening of the cornea due to a regression of the intended central corneal flattening effects caused by a contractile mechanism being initiated during wound healing [61]. Additional problems include mild to moderate irregular astigmatism leading to visual distortion and glare, particularly in patients with more than 8 incisions [20].

Photorefractive keratectomy (PRK) was the first form of laser ablation [91] that permanently alters the refractive power of the eye using an argon fluoride laser with predictable outcomes and is commonly used to treat myopia [47]. In corrective PRK a wound is created via mechanical epithelial debridement and surgical removal of the corneal disc [47], followed by laser ablation of the basement membrane and anterior stroma [10,46], leaving a smooth wound remaining. This encourages corneal flattening as most of the corneal tissue is removed centrally, with less removed around the peripheral area [91]. Initially the technique was favorable over RK as it appeared that following the PRK procedure that corneal mechanical properties are maintained and an increased reproducibility between patients was observed [47]. 

Laser *in situ* keratomileusis (LASIK) or Epipolis LASIK (Epi-LASIK) is currently the prevailing refractive surgery [91]. During LASIK the epithelium is mechanically separated below the basement membrane and just above the bowman’s layer. It is favorable to PRK as the epithelial layer and cells are well preserved and so act as a protective layer preventing exposure to tears and the ablated stroma. This is important as tears contain the growth factors and cytokines associated with fibrotic response and scarring. LASIK surgery also bypasses the pain and extensive visual recovery associated with PRK procedures and can be used to treat higher degrees of myopia [91]. Furthermore it is sometimes favorable to LASEK as it negates the need for potentially toxic alcohol contacting the epithelium and stroma. However it is by no means a perfect technique as sometimes epikeratome flap failure often warrants a conversion to PRK [1,91]

Laser subepitheial keratomileusis* (*LASEK) was originally called alcohol assisted flap PRK as it is a combination of PRK and LASIK techniques [91]. The technique involves the chemical reduction of the adhesion molecules that attach the epithelium to the Bowman’s layer. A marking trephine (cylindrical blade) 8–9 mm in diameter is placed onto the surface of the cornea and a dilute alcohol solution is added to loosen the epithelium [91]. Cold balanced salt solution is used to wash away the alcohol. The corneal mark left by the trephine is dried with a surgical spear to aid delineation (marking of the outline). The epithelium is lifted and peeled back as a complete flap and the surface of the cornea is dried and ablated with an eximer laser. The epithelial layer is then repositioned and a bandage contact lens covers the wound for 3–5 days allowing for re-epithelialzation [91]. LASEK is sometimes preferred by patients over LASIK as it has a lower risk of corneal estacsia (loss of substance and scarring, resulting in a bulging of the tissue) [91] and a faster healing and rehabilitation period [91]. However, LASEK has been associated with patients experiencing pain following surgery. Cold-balanced salt solution and systemic steroid treatments have all been employed to help alleviate this. 

### 5.2. Associated Scar Tissue Formation

It has been reported that RK-induced wounds can differentiate keratocytes into myofibroblatic cells which are responsible for wound contraction [61] and scarring. Light scattering from the radial incisions and/or scars may lead to “starburst” patterns occurring around lights at night, which can make driving hazardous. Mitotic agents including brimonidine and pilocarpine can be used as a temporary treatment as they shrink the pupil, thus blocking light from the peripheral cornea where the incisions are located [20]. Pain following surgery, under- and over-correction, vascularization and endothelial disruption due to the incisions are all attributed to RK surgery. Perforation of the cornea occasionally arises, leading to endothalmitis (inflammation of ocular cavities), epithelial down growth and cataracts [20]. 

Similarly, follow up studies demonstrated that PRK is only analogous to RK as following the primary period of overcorrection; rapid degradation (usually within the first two months) to a relatively constant refraction can lead to corneal scarring, glare and a decrease in visual acuity. The regression in refraction is caused by the normal reaction of the cornea to injury whereby regeneration of the tissue occurs in an attempt to restore corneal integrity following the removal of the anterior epithelium [47]. Laser ablation effectively damages the tissue. The removal of the bowman’s layer causes a pseudomembrane to immediately form (usually within 3–5 days), which covers the ablated area with 3–5 cell layers (a healthy epithelium usually contains 5 to 7 cell layers in humans) [1,79,91]. Activated epithelial cells, fibroblasts and macrophages migrate towards the wound site in order to remove the damage tissue and remodel the affected tissue. The keratocytes beneath the basement membrane undergo apoptosis, typically in the superficial region. Invading inflammatory cells come from the tear film and activated keratocytes (fibroblasts) form new collagen and a proteoglycan matrix, followed by the disappearance of the pseudomembrane [91]. However, in PRK procedures performed on rabbit corneas apoptosis has been observed up to 85 μm into the stroma [10,59]. In PRK studies performed on rabbits, monkeys and humans the degree of scar tissue formation has been linked to the ablation depth, *i.e*., more scarring occurs with greater ablation [47]. The accuracy of the procedure is described as the percentage of eyes within ±1.0 diopters of the intended refraction [47]. This results in a natural predisposition towards an increased accuracy for the improvement of low refractive errors, *i.e*., the larger the attempted correction, the lower the accuracy. 

Considerable variations in subsequent scarring causes light scattering and corneal haze [23] approximately 1 month post surgery, which can increase in severity up to 3 months [91]. The definition of corneal haze varies considerably in the literature possibly due the largely empirical approach of determining success rates. The severity of haze is graded from 0 to 4. A 0 score is an optically clear cornea, where as a 4 indicates an opaque cornea which is visible to the naked eye. Often corneal haze resolves, unaffecting vision, but in more serious cases, results in the loss of unaided visual acuity [91]. A number of treatments have been investigated with the aim to reduce corneal haziness following PRK. The application of corticosteroids is known for their ability to inhibit corneal stromal healing and have been used in PRK experiments to reduce the deposition of scar tissue [47]. Topical steroids are also used to prevent macrophage and lymphocyte intervention in the wound site, reducing collagen deposition by fibroblasts. Anti-metabolites (a metabolite inhibitor) including 5-fluorouracil and mytomycin-C are used to increase the effect of topical steroids as they help to prevent fibrosis. However, toxicity concerns emerge regarding both epithelial and fibroblast cell viability, which can impede epithelialization. In-depth, long-term follow-up studies regarding refraction are infrequent in literature.

### 5.3. Summary

Refractive surgery techniques are ever growing in use and popularity. However, as of yet there are no imminent, continuous follow up studies published for many of the techniques. Existing long-term follow up studies (CK and RK) are discouraging and highlight procedural instabilities and corneal regression [20] such as epithelial hypertrophy is a common problem often requiring further “touch up” procedures [79]. Photoreactive keratectomy and laser *in situ* keratomileusis are being improved but are often criticized for using largely empirical approaches [3]. Three generalized patterns of healing have been noted with regard to refractive surgery techniques. A faint reticular haze occurring in the subepithelial space is observed in type I healing. This usually ceases 1–3 months following surgery. Subepithlial haze does not occur in type II healing, but hyperopia (long sightedness) occurs which often requires additional epithelial debridement or further refractive surgery. In fact, 5%–20% of all patients having had refractive surgery require secondary treatments in order to accomplish a satisfactory result [91]. In type III healing a substantial subepithelial reticular haze occurs which is observed on the cornel tissue, frequently combined with an advance in myopic shift (also a symptom of cataract formation). All keratorefractive techniques cause a refractive change via alterations in corneal curvature [20]. Corneal ectasia (loss of substance and scarring) is an acknowledged development associated with refractive surgeries. Large ablation depths, high myopic treatments, thin remaining stromal thickness and touch-up treatments all increase the risks of developing ectasia. Ideally, photoabalative treatments should not initiate new optical problems. The reduction in corneal thickness often as a result of the surgeries has significant drawbacks concerning intraocular pressure and glaucoma screening and management [91]. In addition, corneal surgery has been criticized for being unpredictable resulting in over or under correction, or the development of antagonism [3]. Such results are predominantly due to the lack of knowledge. Further progress in the understanding of wound healing and post operative pain management are continuous challenges [91]. Appropriate corneal models have the potential to fill the voids in knowledge in order to improve current treatments of corneal disease, which could lead to a reduction in the demand for corneal transplantation.

## 6. Corneal Grafts and Corneal Tissue Engineering

The change in corneal curvature elicited by external surgeries or removal of corneal issue, no matter how small, inevitably provokes a wound healing response. Currently, the only truly successful treatment available for many corneal diseases is corneal allografting. Although the cornea is immune privileged, transplantation carries many risk factors. There are numerous alternatives that are being investigated in order to replace donated tissue [89], or to possibly suppress the demand for donated tissue [93]. Amongst these are xenografts, keratoprostheses and tissue engineered corneas. 

### 6.1. Corneal Allografting

Corneal allografting is often referred to as corneal transplant, keratoplasty or penetrating keratoplasty (PK). The graft replaces the damaged central corneal tissue with healthy corneal tissue from the same species. Unlike other forms of tissue transplantation, corneal allografting are routinely performed without the aid of prior tissue typing or systemic immunosuppressive drugs. The cornea and corneal allografts are often referred to as immune privileged due to its avascular nature [94], however clinicians often object to this proposition as immune rejection is the leading cause of corneal graft failure and remains as a barrier to successful transplantation [94]. Corneal allografts are in fact immunogenic (capable of inducing alloimmune responses) and antigenic (susceptible to antigen specific responses by alloimmune effectors). The rate of corneal graft rejection is variable amongst studies and may be due to differences in patient population, diagnostic rejection criteria and immunosuppressive treatments [95]. The success of corneal transplantation does not compare to the improvements in outcome seen in other clinical transplantation procedures [96], with graft failure most common in patients who have received grafts for conditions other than keratoconus (conical cornea) or stromal dystrophies [96]. The success rates in such patients in considerably lower than the survival of solid vascularized organs, with so far no evidence of improvement [96]. Grafts onto inflamed recipient corneas reject most frequently and rapidly [96]. Once inflamed, a cornea is never the same with regards to immune privileges. Rejection of the donor endothelium is the principal source of transplant failure. Rejection of allograft materials most commonly occurs within 4–18 months post transplantation, but can be seen anytime following surgery [95] sometimes years after surgery [94]. Risk factors that lead to graft rejection involve surgery technique (loose sutures and premature suture removal), the extent and severity of vascularisation in the recipient, the number of regrafts, bilateral grafting, inflammation, glaucoma, dermatitis and dry eye conditions [95]. Alternative approaches are required which account for the unique responses to corneal allografting. 

Ultimately, it is the lack of suitable donor tissue and high graft failure rates that warrant the need for alternative procedures [18,89] particularly in patients where immunological privileges are reduced [96] and additional suitable grafting materials. The successful generation of suitable corneal replacements could reduce the demand on eye bank tissues whilst also aiding in the generation of more effective drug and therapeutic treatments. 

### 6.2. Xenografts

Xenografts are when tissue(s) from one species is transplanted into a different species; for example, a tissue of bovine, porcine or non-human primate origin transplanted to humans [97]. Xenografts have advantages compared to allografts in that it offers a virtually unlimited tissue source and scheduling is impendent of the availability of a human donor [97]. Porcine corneas are commonly used as they have a similar physiology to human corneas and are relatively easy to obtain [98]. The prevailing problem concerning xenografts is interspecies differences leading to graft failure and disease transmission [8,97]. Reports of corneal xenotranplantation models often show different results in different species, so the mean survival time varies from days to months [98], but all eventually fail due to immune response. Glucocorticoids are often used as immune suppressants as they block inflammatory mediator release and inhibit cytokine production. However, more research into immune suppression following surgery is still required as the precise mechanism of corneal xenografts rejection is still unknown [98]. 

Commonly the rhesus monkey is used in xenotransplantation studies as it shares anatomical, histological and immunological responses to humans, thus can act as a predictor of clinical performance. However they may not faithfully represent what would happen if a porcine organ was transplanted into a human subject because non-human primates may develop immunity against human proteins expressed in pigs [98]. Studies have been made looking at the possibility of using porcine corneas for human grafts [8,99]. However the initial results were poor potentially due to the small but significant structural differences between animal and human corneas. In summary the risk of cross-species disease transmission has given the public a poor perception of xenografts; thus this technique will probably never develop further than pre-clinical stage research. 

### 6.3. Keratoprostheses

Keratoprostheses were essentially the first form of artificial corneas developed [6] and offer hope to patients with dry eye conditions such as severe ocular surface disease, chemical burns or corneal vascularization [100]. Surgery is usually only offered to patients with bilateral corneal blindness or those unsuitable for allografting [28]. 

**Figure 5:**
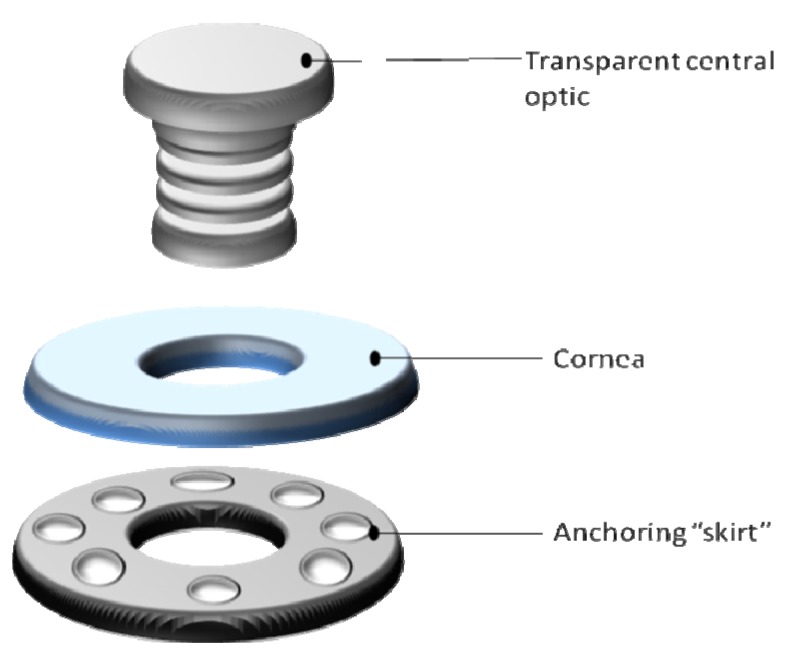
A schematic diagram of an atypical keratoprosthesis

A keratoprosthesis is an acellular artificial implant that ideally should be biologically, mechanically and functionally anchored to the eye tissues without biological and mechanical adverse side-effects [101], permit light transmission in the visible range and protect the retina from UV damage [1]. Materials including glass, fiber glass, silicone, nylon, cellulose [102], poly(vinyl alcohol) (PVA) [101], Darcon fabric [101], Teflon [102], and hydrogels have been examined for the keratoprostheses use. The majority of keratoprostheses are based upon a transparent central optic, surrounded by an anchoring “skirt” that promotes cellular integration from the host [6,28] (Figure 5). A keratoprosthesis that has undergone clinical trials is AlphaCor™ originally known as the Chirilia Keratoprosthesis [103,104]. AlphaCor™ comprises of cross linked poly(2-hydroxethyl methacrylate) (pHEMA), which is one of the most extensively examined materials for keratoprostheses. AlphaCor™ has been implanted into human patients with promising results achieved [104]; however, patient maintenance of the devise is principle to its success post surgery [1]. 

The Boston Type 1 [100] is a commonly used keratoprosthesis in the United States that has undergone clinical trials. It consists of two plates made from poly(methyl methacrylate) (PMMA) with a titanium locking ring to hold the back plate in place. PMMA is the most frequently used material and can be made in UV absorbing forms for intraocular lenses. It can be shaped and preserves clarity whilst having low toxicity [1], but its rigidity and hydrophoboicity lead to poor diffusion.

The procedure to implant a keratoprosthesis is complicated and to date, clinical use of keratoprostheses has been little. There still needs to be a great deal of research and improvement made in this area before keratoprostheses are used to replace donated corneas in corneal transplants [1]. Despite huge advances in the technology used, biocompatibility is still an issue [102,105] as many of the materials are non-cell adhesive so improvements need to be made to modify the ability of corneal cells to adhere and migrate over the surface [6]. Biological compliance and attachment of prosthetic devices relies upon the ability of stromal cells to migrate into the device and deposit matrix [1]. Epithelization is one of the most demanding features that need to be considered when producing a keratoprotesis, as often the act of blinking can shear away the epithelial cells on the transplanted prostheses [1]. Keratoprothesis devises are generally more successful when the descements membrane is intact [1] however; severe eye conditions have often damaged or destroyed it. Failure of a prosthesis to correctly anchor can lead to extrusion or protrusion of the implant [28,102] which in turn can affect mechanical strength [8,106]. 

### 6.4. Tissue Engineered Corneas

Tissue engineering (TE) can be described as “the production of biological or semi-synthetic living tissue for use as replacement tissues for damaged or diseased tissues” [107]. Technically speaking, it is the generation of a tissue by seeding isolated, specific cells or stromal cells into or onto a template (often referred to as a scaffold) and culturing it in a dynamic environment with the aim to eventually form a tissue which mimic the morphological, physiological and biochemical properties of the natural tissue as closely as possible [107]. The idea of a bioengineered cornea has emerged with corneal counterparts being assembled from corneal cell lines [1]. With respect to corneal tissue engineering there are many challenges that need to be addressed, not least the fact that the structure of the cornea is unique and difficult to replicate. Two fundamental challenges in corneal TE are the maintenance of phenotype in isolated stromal cells and the replication of the collagen architecture. If both factors are not satisfied, the result is often a regenerated tissue mimicking that of scarred native tissue. Thus far, advances in the development of a fully bioengineered cornea are still at an analytical stage [1]. At present when attempting to construct a TE-cornea tissue strength and transparency are the main areas of focus as these are lacking in current models [108]. 

When constructing a tissue-engineered cornea, the choice of scaffold material is vital [109,110] in order to enable the cells to behave and form the complex arrangement as they would in *in vivo*. Amniotic membrane, collagen, fibrin, cross-linked fibronectin-fibrin gels, gelatine-chitosan, collagen-glycosaminoglcan blended nanofibrous scaffolds, contact lenses and thermoresponsive polymers are amongst the vast assortment of materials investigated as suitable corneal substrates [111]. However, as of yet few have achieved clinical approval [111]. The list of requirements for a suitable scaffold is extensive, as they must be biocompatible [93], optically transparent, strong as to withstand manipulation in culture, potential suturing, irrigation and handling in surgery. The material also needs to be flexible to take the shape of the eye and lay flat on the surface. It needs to be able to be produced easily with consistent quality, preferably at high speed and low cost [112], have controllable biodegradability or bioresorbability enabling cultured tissue to replace the scaffold material [110] and have the spatial architecture of the native tissue. There are various arguments as to whether natural or synthetic biomaterials are more suitable. In general, the properties of native soft tissues are not easily duplicated by synthetic materials [113], as the cells do not recognize the surfaces, so do not attach [110]. *In vivo* cells thrive on ECM comprised predominantly of collagen, glycoproteins, proteoglycans, laminin and fibronectin. 

In order to successfully achieve a tissue engineered corneal substitute the ability to successfully culture epithelial, keratocytes and endothelial cells that will proliferate in culture conditions whilst retaining most of their *in vitro* characteristics is required [56]. Currently we and other groups recognise that the culture of pure corneal cells is problematic [114], and that the optimal culture conditions of the isolated cells need to be defined and optimized. It has already been established that keratocytes differ in a variety of ways from fibroblasts and myofibroblasts. Cell morphology, proliferation and the expression of gene products are identifiable as being significantly different [57]. Phenotypical changes occur when a cell becomes fibroblastic and it has proven difficult to reverse such changes when attempting to culture keratocytes *in vitro*. Studies have suggested that it is possible to culture keratocytes in culture whilst retaining their *in vivo* phenotype [57]. Previous investigations have also claimed that it is possible to de-differentiate corneal myofibroblasts back to their fibroblast phenotype [57]. However, the main challenge still remains for researchers to differentiate fibroblasts to a keratocyte phenotype. The successful differentiation would be hugely significant with regards to corneal transparency *in vivo* and *in vitro*. Transparency can be restored in corneal scar tissue *in vivo* suggests that fibroblasts are capable of returning to the keratocyte phenotype and replacing the disorganized ECM of the scarred tissue [57]. The suppression of scar tissue formation is the general strategy employed for functional corneal tissue formation. *In vitro* manipulation of chemical, topographical and cellular environments may enable us to mimic the conditions required to do this.

#### 6.4.1. Manipulation of Chemical Cues

Chemical cues supplied by culture media and supplementation can influence cell phenotype *in vitro*. This is not specific to corneal cells but is true for most cell types. Successful culture of keratocytes is imperative for restoration of corneal transparency in stromal equivalents. Many studies demonstrate that when cultured in serum-free media corneal stromal cells maintain a quiescent cell phenotype associated with keratocytes [55,57,62,115]. However quiescence behavior alone is not sufficient in determining keratocyte phenotype as it is not unique to the keratocyte phenotype [55]. Often, the quiescent behavior of cells due to removal of serum is followed by apoptosis [67]. Thus supplementation is required in media that can promote cell growth and proliferation of keratocytes without encouraging fibroblastic differentiation [67]. 

Studies performed using primary cultures of bovine keratocytes [57] have shown that the presence of serum causes the cells to turn fibroblastic [57]. The addition of TGF-β without serum can also cause the same result [55,57]. It was hypothesized that the removal of serum would cause the cells to differentiate towards a keratocyte morphology. Following 8 days culture in serum free media the cells did in fact return to a morphology that was keratocyte-like in appearance and it was also suggested that the surrounding ECM was also restored to a keratocyte profile. Unfortunately the levels of aldehyde dehydrogenase (ALDH_3_)-a cytoplasmic protein vital to corneal transparency did not return to levels expressed in keratocytes. This promising study suggests that the partial restoration of the keratocyte phenotype following a fibroblastic transition is possible. Unfortunately the addition and removal of serum from media is not enough to fully restore the full phenotypical profile of a keratocytes and more complex biochemical cues still need investigation. 

The list of biochemical components included in “keratocyte media” in current literature is extensive and often contradictory. Acetycholine, insulin [56], vitamin C [13,108] growth factors [56] and cytokines [55] are but a few amongst many supplements that have been investigated when attempting to obtain a “pure” proliferating culture of keratocytes. It should also be noted that often studies that look at the effect of biochemical cues are performed under simplified two dimensional (2D) tissue culture conditions. Interactions between the keratocytes and competing environmental stimuli are not considered [55]. Multiple growth factor and ECM signals may be important to keratocyte differentiation. Thus a 3D environment may be required in order to more closely mimic the environmental cues required to successfully culture and between keratocytes and its sub-types. 

#### 6.4.2. Utilizing Topographic Cues

Native corneal tissue primarily consists of collagen type I. Collagen type I hydrogels commonly used in tissue engineered tissues as scaffolds formed by conventional thermal gelation often have poor mechanical strength and lack organization, which is contrary to the characteristics of native stroma [116]. Naturally, keratocytes grow in an orthogonally arranged architecture, following the organization of the collagen fiber bundles. Much research has focused upon mimicking this architecture in both 2D and 3D cultures. Contact guidance techniques have been extensively researched as they are able to affect many cell characteristics including orientation, morphology, differentiation and secretion of ECM proteins [89]. It is the material composition and more specifically the 3D nano- and microscale structure (the mesostructure) of bioartificial constructs that are pivotal to their success [117]. Micro- and nano-patterned surfaces, magnetic alignment and electrospinning techniques are amongst a variety of techniques utilized in order to achieve this.

#### 6.4.3. Micro- and Nano-Patterning

Micro- and nano-patterned surfaces have been thoroughly investigated when attempting to mimic the corneal fibrillar arrangement [89]. They are often manufactured via the use of templates with well defined groove widths and depths into which cells with and without matrix materials are added [89]. The patterned surfaces effectively restrict random cell growth via the incorporation of either physical or biochemical barriers. Orientated deposition of ECM components is capable of reinforcing the substrate in a given direction, which enhances the global mechanical properties of the original construct [89]. Vrana *et al.* [89] demonstrated the ability to align both cultured stromal and retinal pigment epithelial cells onto micro-patterned collagen films. The cultured stromal cells showed increased cell and ECM orientation and elongated morphology for up to 7 days culture duration. However, following 3 weeks culture the cell orientation decreased. This was attributed to the fact that the cells were able to adhere to the inclined walls of the micro-patterned surfaces following proliferation and thus filled the space at the base of the aligned channel. The epithelial cultures however, failed to secrete significant ECM proteins and showed reduced proliferation. Fibroblast and epithelial ECM secretions are different, but it has been demonstrated that surface patterning can impede extensive cell-cell contacts characteristic of epithelial cells, which alters proliferation capabilities [89]. 

#### 6.4.4. Magnetically Aligned Collagen

Magnetic fields have been utilized in an attempt to create a stromal like-scaffold made up of multiple intermeshed orthogonal layers of orientated collagen I fibrils [26]. The use of magnetic fields to induce collagen orientation is advantageous in that it is non-destructive [26]. It has been reported that molecules of collagen can be assembled into orientated fibrils via the application of a magnetic force [26]. In brief, this can be achieved by loading an aliquot of collagen into a shallow sample holder and positioning it horizontally in the central region of a split coil superconducting magnet and increasing the temperature from 20 to 30 °C for approximately 30 min. The collagen molecules assemble into orientated fibrils perpendicular to the applied field and transform into a viscous gel that is stable and orientated after the magnetic field is removed. The orthogonal arrangement is achieved by adding more collagen and repeating the process at an altered angle, in a layer-by-layer approach. Human keratocytes can then be successfully seeded onto the aligned surface of the gel where they then become uniformly aligned along the collagen fibril direction [26]. 

Although the author claims that the orthogonal scaffold produced using this method are structurally similar to a native stroma they are flawed in that they are not highly transparent. Lack of transparency could be due to the fact that the fibril diameter is much greater than would be found in a native cornea, approximately 100–200 nm, in comparison to 20–30 nm. The addition of proteoglycans has been shown to reduce fibril diameter thus increasing transparency [26], but unfortunately no systematic study has been performed to fully understand and exploit this phenomena. Unfortunately fibril diameter cannot be regulated when using such a technique.

Despite the claims of this promising technique being used to form the basis of a corneal implant for corneal regeneration, there has been very little literature published since for *in vitro* cornea applications. There is also conflicting evidence suggesting that the application of strong magnetic forces can in fact impair cell function and viability [26,118]. It should also be noted that the keratocytes were only added to the collagen construct after the gel had been aligned and set as the technique is non-sterile. So it is questionable as to whether the cells are able migrate through the full thickness of the construct as they would in a native cornea. 

#### 6.4.5. Electrospinning of Nanofibers

Electrospinning is a century old process that is able to produce continuous fibers from the submicron diameter down to the nanometer diameter [119]. These fibers can then be arranged to recreate the microstructure and arrangement of the collagen fibers in a natural cornea. Typically collagen type I solutions are combined with a synthetic polymers such as polyethylene oxide (PEO) or 1,1,1,3,3,3-hexafluro-2-propanol (HFP) as a solvent. However, HFP is difficult to work with and toxic so it could be argued as to whether it would be a suitable or safe material for tissue engineering applications [112]. No one as of yet has created aligned type I fibers on a small enough size scale comparable to those in the native cornea [18]. It has been shown in 2D culture conditions that keratocyte populations are able to align in multiple adjacent layers arranged 30–90° to each other [108,120]. Wray *et al.* [18] and Wu *et al.* [121] have previously used aligned collagen and poly (ester urethane) urea fibers fabricated by electrospinning, to grow corneal stromal cells. However, these studies were limited to a single dense nanofiber sheet and did build the full organization similar to that of the native tissue. In order to generate a functional corneal tissue, a 3D environment with defined topographical cues throughout a 3D construct is required [67]. 

Wray and Orwin [18] report that they have managed to recreate smaller fibers without the use of toxic solvents or polymers which support the growth of corneal fibroblasts and encourage the cells to align along the fibers. However, they did not comment on cell morphology, culture duration, or if the fibers induced differentiation of the cells. Only corneal fibroblasts were reported to grow along the fibers in this study which as previously mentioned are not actually found in the native healthy cornea and are significantly easier to cultivate, expand and manipulate in culture than cells native to the healthy cornea. It has not been determined how the fibers affect transparency of the aligned fibrous scaffolds neither. 

It has already been shown that the three main corneal layers can be recreated *in vitro* using collagen-based scaffolds [5]. However these models are still lacking the highly organized 3D collagen based architecture that is present in a native stroma [26]. Thus the biomechanical and optical properties of the cornea models are compromised as it is the collagen fibrils that reinforce biological materials [21]. By improving collagen orientation so that it is more organized, as in a native cornea, would result in improvements in both optical and mechanical properties and so is an area that future research needs to pay more attention to. The ability to construct a scaffold that has orthogonal lamellae of aligned collagen fibrils is desirable in the development of tissue-engineered corneas [26] and has yet to be successfully achieved. It is also interesting to note that the majority of corneal diseases result from disruption of the highly organized collagen fibril arrangement in the stromal layer [3]. Thus if a more accurate corneal model that incorporated the corneal fibril organization into their design were produced then it could be used by corneal surgeons to more accurately predict the outcome of surgical procedures. Scaffolds able to mimic the native corneal organization may be beneficial as they create a strong tissue substrate that may accelerate the healing process, aid orientation of the subsequent secreted ECM and increase the tensile strength [67] that is often lacking in artificial constructs. When used in combination with chemical manipulation of culture conditions the use of aligned electrospun nanofibers arranged into orthogonal 3D layered constructs has also been shown to change the cell phenotype of contractile stromal fibroblasts towards a quiescent keratocyte lineage, which display a more elongated dendritic morphology and express genetic markers characteristic of the native keratocyte phenotype [67]. 

The incorporation of electrospun nano-fibers into corneal tissue engineering is a technique that is still in its infancy and evidently needs a lot more research before it can feasibly be used as a way of mimicking fibril orientation in *in vitro* corneas. It is however a potential avenue for future exploration and improvement. 

#### 6.4.6. Co-Culture Approaches

There are numerous cell based approaches that have been reported, all with the similar aim to manipulate the cells to create their own ECM so that they behave the way that they would in a natural cornea. There is a great deal of information in the cells themselves that needs to be understood as is the cells that contribute to the transparency and strength of the cornea. The majority of culture systems for corneal tissue separately culture epithelial cells, keratocytes/fibroblasts and endothelial cells as mono-layer cultures [122]. 2-Dimendional (2D) monolayer cultures have been used to investigate the way that various exogenous growth factors can be used to regulate growth, differentiation and function of corneal cells [123]. However, monolayer culture fails to mimic the *in vivo* physiological environment and lack the 3-Dimensional (3D) physiological environment found *in vivo* tissues and so are often criticised with regards to their limited application to the *in vivo* situation [124].

Most tissues consist of more than one cell type and it is the organisation of the cells within the tissue that is essential to normal development, homeostasis and in the case of corneal tissue, transparency. *In vivo,* epithelial cells are in close contact with keratocytes [125,126] in the stromal layer; they are connected both anatomically and functionally [77]. Co-culture studies aim to recapture this cellular anatomy and functionality. It has been suggested that that epithelial cells proliferate more actively and are better differentiated in co-culture systems with keratocytes and endothelial cells than when in separate cultures [122] and their proteoglycan synthesis is significantly less in monocultures than when cultured as a whole intact cornea or in a co-culture system [127]. This may be due to the production of as-of-yet unknown (combinations of) growth and differentiation factors that affect the growth and proliferation of the cells [122]. It has been suggested that growth factors and cytokines secreted by epithelial cells regulate keratocyte cell function and *vice versa* [46,65] in a reciprocal bidirectional manner that aid and stimulate normal migration and the secretion of proteoglycans and glycoaminoproteins in a simultaneous, highly coordinated manner which changes dependent upon development, homeostasis and wound healing; although direct cell-cell communications do occur in some situations [128] that are vital to cell response [54]. This interaction is critical in *in vivo* wound healing and may have important connotations for *in vitro* co-cultures. Differentiation in the cornea involves multiple growth factors, cytokines and extracellular environmental signals [55], thus a 3D environment is often required to mimic the extrinsic environmental as well as the intrinsic cellular cues necessary to successfully culture and differentiate stromal cells and their subtypes. *In vivo* it is the interactions between the keratocytes and adjacent epithelial cells that are vital to maintaining tissue homeostasis and transparency. Cytokine-mediated cellular communications are very complex and although the cell-cell cross talk is not fully understood, the communication that occurs is unlikely to be unidirectional [77]. The reciprocal interactions are fundamental to early development and extend into adulthood in maintaining organ function [127]. Although much work still needs to be done, it is apparent that cell-cell communication is fundamental in ensuring that the cells in the stromal layer maintain their keratocyte phenotype which is vital to the corneas development, homeostasis and response to environmental stimuli including injury and infection [128]. Specifically, the cellular communication aids in the restoration of activated keratocytes (fibroblasts) back to a quiescent keratocyte phenotype following injury, thus restoring tissue transparency which avoids excessive scarring that is detrimental to vision. 

Co-culturing of cells is becoming an increasingly popular method to create a full thickness cornea as it has the advantage of cell-cell interactions occurring, thus mimicking normal call behavior in a natural cornea. In co-culture systems 2 or more cell types are bought together within the same culture environment, enabling them to interact and communicate which can act as a very powerful *in vitro* tool allowing cellular interactions and cell function to be studied [129]. For example, when treating limbal stem cell deficiency (LSCD); a condition characterized by the loss of epithelial stem cells at the limbus, success is dependent upon the proliferative capacity of the transplanted epithelium over time. This in turn is determined by the origin and phenotype of the underlying stromal cells [48]. The influence of cellular interactions is of particular interest to tissue engineers because the tissue formation of one or all cell types can be regulated by simulating the natural physiology and differentiation of cells. Previous co-culture studies have displayed that a collagen gel matrix is essential for epithelial cell growth, and maintenance of structural differentiation [123] and that additional stromal cell cultures can influence the differentiation of epithelial cultures and improve stratification of epithelial cells [111].

When developing co-culture models, it is important to balance the ability to observe, measure and manipulate cell behavior when constructing *in vitro* 3D environments [129]. For example, *in vitro* corneal tissue has been reconstructed previously [122,123] comprising of epithelial, stromal and endothelial cells in a 3D collagen matrix. Unfortunately the level of complexity involved deemed it unsuitable to accurately monitor and measure proliferation and differentiation in the component cells. Thus, often simplified models are required. These include the use of conditioned media [130], transwell cultures which utilize a semi-permeable membrane [65,131] and direct explant cultures. 

Hibino *et al.* [130] have utilized the use of epithelial conditioned media to investigate cell-seeded collagen gel contraction. Their results demonstrated that the addition of epithelial conditioned media accelerated construct contraction. However, it should be noted that the rabbit keratocytes used were of high passage number (P8) and were cultured in up to 12.5% FCS and that the epithelial cells were cultured in the presence of 15% FBS; thus the stromal cells were likely to be of a fibroblast/myofibroblast phenotype. Corneal wound healing can be stimulated by various exogenous cytokines [132] and many studies including the use of conditioned media, have been performed to help understand the underpinning pathways of intracellular signaling that control growth, differentiation and cell apoptosis. In doing this we could potentially identify specific intracellular targets for drug development that could effectively bypass dysfunctional cytokine receptor control for growth, differentiation and apoptosis [130].

Nakamura *et al.* [65] demonstrated in a co-culture system that injured epithelial cells secrete soluble factors able to cross a membrane impermeable to cells which stimulate corneal fibroblast proliferation, myodifferentiation and matrix contraction. *In vivo* epithelial-stromal interactions mediate fibrotic or a regenerative response, thus correct manipulation of culture conditions may facilitate the ability to control and initiate the cornea’s regenerative capacity on demand in *in vitro* cultures. Thus, it can be surmised that uninjured epithelial cells can likewise secrete soluble factors which can potentially influence fibroblasts to return to their pre-injury phenotype, *i.e*., a keratocyte. 

Nakazawa *et al.* [131] have performed co-culture studies by the use of insert dishes and companion plates to study epithelial-stromal and endothelial-stromal interactions. Stromal seeded collagen gels were cultured on the insert dish with either epithelial or endothelial cultures on the companion plate. The method is convenient in that it allows for the individual cultures to be easily separated and analysed in terms of growth and proteoglycan synthesis. Interestingly, they found that in comparison to mono-cultures, cell growth was not stimulated in any cell type in either epithelial-stromal or endothelial-stromal cultures. However, this is not consistent with the work of others [127,133,134]. The inconsistency was not determined in the study, although may have been due to the lack of successful adhesion of the epithelial and endothelial cells to the companion plates and/or the fact that the media was changed daily, which could reduce the stimulatory effect of the soluble cytokines released into the media. Additionally, the dispase treatment used to separate the cells from the tissue may have damaged the adhesion sites of the cells. However, it was noted in the previous studies that the proteoglycan content was significantly altered in the co-culture studies; stromal and epithelial cells stimulated proteoglycan synthesis by each other [127]. Analysis via proteoglycan radiolabelling demonstrated that epithelial and endothelial cultures had different effects on the stromal cultures, suggesting that *in vivo* proteoglycan synthesis in a normal cornea is defined by a careful balance between the antipodal actions of the epithelial and endothelial layers [127].

Much of the existing literature regarding corneal co-culture is concerned with epithelial-stromal culture, with the emphasis being on the effect that the additional stromal cells (often fibroblastic in phenotype) will have on the epithelial culture. This may be due to the interest in treating limbal epithelial deficiencies and epithelial wounds. However, often corneal disease and injuries (such as chemical burns and Stevens-Johnson Syndrome [34]) penetrate deeper into the stromal layer so it is important to also consider the effects that epithelial cultures will have on the corresponding stromal culture. 

A greater understanding of stromal-epithelial-endothelial interactions and what mediates them by utilizing co-culture approaches offers great pharmacological potential in the regulation of corneal wound healing, with the potential to treat corneal diseases and injury whereby such interactions are vital [77]. As the different healing mechanisms, contributing factors and their potency become known, we are able to utilize this information in order to accelerate and enhance the wound healing process [135]. However, despite the accomplishment of culturing epithelium, stromal cells and endothelium onto/in collagen based hydrogels there is still no tissue engineered corneal substitute suitable for clinical use. The production and culture of viable human endothelium is a prevalent factor, but the main problem still involves replication of the appropriate corneal stromal architecture [1]. 

#### 6.4.7. Growth Factors

Growth factors and cytokines play a pivotal role in the wound healing process. In order to generate transparent corneal constructs via tissue engineering techniques a feasible strategy to control keratocyte activation and myofibroblast differentiation is to utilize the use of various growth factors. Many studies have been performed using this strategy, however they are often contradictory, and dose dependent or incomplete. For example, it has been shown that FGF may be used to stimulate epithelial coverage, but few studies have been performed to accurately verify this. FGF has also been shown to reduce the expression of α-SMA in 3D *in vitro* cultured keratocytes [68]. However, in 2D cultures FGF has caused fibroblastic differentiation [49]. The contradictory lack of stress fiber formation and change in matrix composition when in 3D cultures compared to 2D cultures suggests the FGF stimulation may be sensitive to the surrounding ECM. Additionally, discrepancies in FGF studies may be due to the use of non purified FGF from bovine brain or human placenta sources which will lead to controversy regarding the appropriate dosage [136]. Recombinant or purified FGF may be more appropriate to dose-response studies [136]. Discrepancies in the concentrations or combinations of growth factors used can affect the overall cellular response.

There are 3 different isoforms of TGF-β in humans [40,41,69,70,71,73,75] and it becomes strategically obvious that we have to treat the TGF-β isomers differently. The *in vivo* affects of these growth factors have been mimicked *in vitro* via exogenous treatment with TGF-β1 and -β2 with the effects emphasized at higher cell densities [68]. The roles of each of the isoforms are distinct and differ from each other [71]. Although their exact roles still eludes us, it has been suggested that optimal treatment may rely upon selective inhibition of more than just one of the isoforms [72]. Inhibition of TGF-β1 and -β2 through the use of exogenous agents (such as Smads) and antibodies can reduce and eliminate myofibroblast differentiation and fibrosis *in vivo* and *in vitro* [72,74]. TGF-β neutralizing antibody can prevent and block this action and has been used in previous collagen hydrogel co-culture studies to prevent hydrogel contraction caused by the release of soluble factors [65]. On the other hand, stimulation with TGF-β3 has been shown to yield corneal stromal models with non-fibroblastic characteristics with ECM properties that more closely mimic the native stromal ECM [71]. Hence, the use of TGF-β3 has been speculated for use as a therapeutic agent. TGF-β3 is thought to stimulate and activate different signaling molecules to TGF-β1 and TGF-β2 [71]. This results in a differential TGF-β function that inhibits type III collagen synthesis which is usually significantly upregulated and more abundant in wounded, fibrotic corneas [74]. Addition of TGF-β3 does not cause the upregulation of -α-SMA as occurs with the TGF-β1 and -β2 isoforms [71]. In agreement with these studies, Shah *et al.* [137] have also demonstrated that an increase in TGF-β3 production relative to TGF-β1 and TGF-β2 results in a scar-free phenotype. 

During TGF-β signal transduction a combination of proteins known as Smads are superficially initiated by TGF-β super family members [45]. Following TGF-β receptor binding, serine-threonine kinase receptors are activated which bind to receptor-activated Smads (R-Smads), Smad-2 and -3. These are then phosphorylated to form a complex with Smad-4. These complexes transfer into the nuclei whereby they organize transcription of the target gene [45]. Smad-7 acts by disrupting Smad-2 and -3 activation via competitive binding to the TGF-β receptors, thus inhibiting TGF-β signal transduction. Additionally Smad-7, an inhibitory Smad can also prevent TGF-β signal transduction [45]. TGF-β2 has been established as the primary mediator of fibrosis and scarring. Blocking TGF-β2 using Smad-7 may reduce the keratocyte activation in response to injury thus preventing fibroblast and myofibroblast differentiation. It acts in the nucleus, altering the formation of TGF-β induced functional Smad-DNA complexes [45] and has a therapeutic potential for reducing excessive fibrosis and scarring. 

The effect of using PDGF is frequently associated with the use of serum in media which induces fibroblastic response. However serum contains a vast assortment of undefined factors as well as PDGF that may also contribute to the fibroblastic differentiation. When used in serum-free media, PDGF has been shown to facilitate the elongation and generation of dendritic procedures of stromal cells [17] and cause little matrix deformation. This allowed for keratocyte repopulation of the corneal stroma without disruption to the fibril architecture, thus reorganization of the surrounding matrix did not occur. PDGF encouraged more directional progression during migration without the production of stress fibers. The use of Rho kinase has had similar effects. Rho kinase belongs to a family of serine-theonine kinases, associated with regulation of shape and movement of cells. It controls the large traction forces generated by fibroblasts as they migrate. Inhibition of Rho kinase using Y-27632 inhibitor has been shown to alter the migration capability of stromal cells [17].

IGF has been used *in vitro* to stimulate keratocyte growth and proliferation and induces the elongation of the keratocyte dendritic processes [68] required for cell migration. Its inclusion in culture media can help to support corneal structure and may facilitate a regenerative wound healing phenotype [68]. Like EGF, KGF stimulates epithelial cell growth both *in vivo* and *in vitro* [40]. The exogenous *in vivo* addition of KGF to rabbit eyes following epithelial injury has shown to have growth promoting effects to epithelial cells [138]. It is thought to work by stimulating and increasing the mitotic activity of limbal epithelial stem cells in the regenerating cornea. Topical KGF treatment may have clinical potential in the treatment of epithelial disorders, in particular those requiring stem cell differentiation [69] as, unlike many other growth factors, it does not affect cells of mesoderm origin and thus does not induce neurovascularisation of the cornea as a side effect [138]. 

*In vivo* cytokines and growth factors are involved in complex feedback mechanisms whereby the activation of their receptor in a target cell can stimulate the production of additional growth factors and so on. This cascade affect promotes cross-talk to the original cell in a reciprocal epithelial-stromal interaction [46]. Thus when attempting to understand and manipulate the use of growth factors to control cell phenotype in tissue engineered constructs and/or a wound healing outcome (*i.e*., fibrosis or scarring) the use of single growth factors is not enough. We must first unpick and understand the intricate mechanisms and intricate combinations of growth factors will be needed to provoke the desired response. In order to determine an environment that stimulates organized keratocyte migration and generation a suitable model system is needed that will allow for stromal cells to be studied in their natural, uninjured state. 

### 6.5. Summary

The design of any tissue replacement requires a comprehensive knowledge and understanding of the capacity of the native tissue [1]. There have been compelling advances in the development of synthetic corneal replacements and culture of human corneal cells onto and within supporting natural substrates. However, the gross results of these developments are poor and there are currently transplantable tissue engineered corneal equivalents to date [1,89]. There are currently only 2 clinically applicable synthetic cornea replacements (AlphaCor^TM^ and the Boston Type-1 keratoprothesis [1]) and as of yet no viable tissue engineered corneas for treatment of end stage corneal diseases. Mimicking the complex organization of the corneal architecture is a current stumbling block. Two specific hurdles in particular need to be elucidated before appropriate tissue engineered corneas can be accomplished: (i) replacement or replication of the ultrastructure of the ECM; (ii) production of a dependable supply of untransformed proliferative corneal endothelial cells. Additionally lack of tensile strength to permit surgical manipulation and attachment of the corneal equivalent along with the correct formation of appropriate surface curvature [34] are problems that need addressing. Once these are solved storage and transport of the corneal equivalents will need to be reviewed. 

The capacity to successfully culture corneal stromal cells is progressing and the ability to determine differentiation state, contractile behavior and secretion of matrix components via manipulation of factors including but not limited to TGF-β and ascorbic acid derivatives are all encouraging. Commonly used reconstituted type I collagen gels are unlike the real stroma (with the exception of containing type I collagen and stromal cells) Controlling the assembly and/or remodeling of the collagen ECM is impediment as the principle functions of the cornea (strength, transparency refraction) are all directly connected to the nanoscale organization. The utilization of collagen films and nanoscale aligned structures in order to guide stromal cell migration and deposition of collagen and ECM is bringing researchers closer to achieving this goal.

## 7. Conclusions

The cornea is an excellent model for studying wound response with regards to cell phenotype due to the unique homogenicity of the cell types that reside in the defined cell layers and the remarkably organized tissue architecture that if disrupted has immediate and obvious effects. Following injury or disease, the cornea is capable of restoring full function via a regenerative approach and one of the most demanding challenges in corneal biology is in the assistance of tissue repair via *regeneration* as opposed to *fibrosis*. The interaction between the corneal stroma and the adjacent epithelium is known to have a role in wound healing mechanisms although the exact mechanism is unknown. Derivations in its barrier function render it permeable to cytokines and growth factors which can facilitate an activated fibrotic response and scarring. Scar tissue alters the constituents of the original ground substance of the cornea including the proteoglycans, collagen filaments and various proteins via dynamic feedback mechanisms. The extent of damage or injury is known to have an effect on these mechanisms and may dictate whether the healing response is regenerative or fibrotic. Thus recognizing the diverse epidemiologies of common corneal injuries and diseases is of great significance to corneal research and medicine as it may aid in devising more appropriate treatments and preventative measures. Many of the existing treatments fail to cure the underlying cause, are not cost effective and can only alleviate or treat the secondary symptoms of disease. More knowledge of the underpinning mechanism that leads to scar formation and permanent opacities will aid in treatment. 

Corneal surgery techniques and procedures are widely used to improve the refractive properties of the cornea usually via an alteration to the corneal curvature. They are often criticized though for their unpredictability which results in over or under correction, astigmatism, ectasia and scarring. Further knowledge of wound healing following surgical procedures, management of post operative pain and more effective methods of controlling scar formation are continuous challenges. Appropriate models may aid in this challenge. 

Currently, the only truly successful treatment available for many corneal diseases is corneal transplant. However, the lack of donor tissue and relative high graft failure rates warrant the need for alternative procedures [18,89]. Tissue engineered corneas provide a promising solution in comparison to xenografts and keratoprotheses. Nevertheless, there is still no clinically available tissue engineered substitute available to date and tissue engineered corneas are currently limited to *in vitro* applications [93]. The production of a viable endothelial layer is a prevalent factor although replication of the stromal architecture and maintenance of stromal cell phenotype are still the biggest obstacles. Efforts have been made to mimic such architecture although still fail to meet the nanoscale arrangement. Concerted research efforts by multi-disciplinary research groups are ever adding to our knowledge of the complex systems required to restore and enhance vision in both *in vivo* and *in vitro* models. An accumulation of this knowledge will bring us closer to achieving this goal.

## 8. Perspectives

The control of scar tissue formation is undoubtedly of significant relevance from both clinical treatment of corneal diseases and corneal regeneration perspectives. The central challenges are the control of stromal cell phenotype and the organization of stromal ECM. Tissue engineered corneas could be super *in vitro* models allowing us to accumulate the knowledge of precise cytokine and growth factor mechanisms involved in fibrotic and regenerative healing via the addition of specific growth factors and chemical cues individually or in combination. Corneal research and tissue engineering techniques need to be tailored to fulfill the clinical pull and demand. Understanding the wound healing mechanisms and what mediates them will determine and target specific exogenous factors that can be used to inhibit a fibrotic response thus aiding drug development and corneal disease treatment. The future direction of corneal tissue engineering is to further define the complex cell-cell and cell-matrix cross talk that occurs amongst the different cell types during the wound healing cascade. Defined 3D co-culture conditions and application of nanotechnology for stromal ECM reconstruction will allow us to build up more complex corneal models and echo *in vivo* scenario. Smart tissue engineering constructs provide a platform to delineate to impact of individual or combinatorial molecules. This transferrable knowledge will be beneficial for treating corneal disease and injury, in preventing or alleviating corneal scarring for clinical applications and encouraging corneal healing to adopt regenerative healing mechanisms as opposed to reparative fibrotic responses. 
